# Blood pressure and cardiovascular risk in adults with non-proteinuric chronic kidney disease without diabetes

**DOI:** 10.1038/s41440-026-02801-7

**Published:** 2026-09-10

**Authors:** Yuta Suzuki, Masachika Nishikawa, Hidehiro Kaneko, Akira Okada, Toshiyuki Ko, Katsuhito Fujiu, Norifumi Takeda, Hiroyuki Morita, Naoki Nakagawa, Tatsuhiko Azegami, Kaori Hayashi, Takashi Yokoo, Koichi Node, Hideo Yasunaga, Norihiko Takeda

**Affiliations:** 1 The Department of Cardiovascular Medicine, The University of Tokyo, Tokyo, Japan; 2 Center for Outcomes Research and Economic Evaluation for Health, National Institute of Public Health, Saitama, Japan; 3 The Department of Advanced Cardiology, The University of Tokyo, Tokyo, Japan; 4 Department of Prevention of Diabetes and Lifestyle-Related Diseases, Graduate School of Medicine, The University of Tokyo, Tokyo, Japan; 5 International University of Health and Welfare, Tokyo, Japan; 6 Division of Cardiology and Nephrology, Department of Internal Medicine, Asahikawa Medical University, Asahikawa, Japan; 7 Division of Nephrology, Endocrinology, and Metabolism, Department of Internal Medicine, Keio University School of Medicine, Tokyo, Japan; 8 Division of Nephrology and Hypertension, Department of Internal Medicine, The Jikei University School of Medicine, Tokyo, Japan; 9 Department of Cardiovascular Medicine, Saga University, Saga, Japan; 10 The Department of Clinical Epidemiology and Health Economics, School of Public Health, The University of Tokyo, Tokyo, Japan

**Keywords:** Hypertension, Chronic kidney disease, Cardiovascular disease, Epidemiology, Implementation hypertension

## Abstract

We investigated the association between blood pressure (BP) and the risk of cardiovascular disease (CVD) in individuals with non-proteinuric chronic kidney disease (CKD) without diabetes. Using a large-scale nationwide administrative claims and health checkup database in Japan, we identified 287,238 non-diabetic adults with an estimated glomerular filtration rate <60 mL/min/1.73 m^2^ and negative/trace proteinuria. We examined the association between BP and CVD events by modeling BP both as a continuous variable and as a categorical variable according to the 2017 ACC/AHA guideline classification. The primary outcome was a composite of myocardial infarction, stroke, heart failure, and atrial fibrillation. Multivariable Cox regression analyses showed that higher systolic BP (SBP) was associated with increased CVD risk (hazard ratio [HR] 1.05 per 10 mmHg; 95% CI 1.04–1.05). Compared with normal BP, the HRs were 1.03 (95% CI 1.00–1.07) for elevated BP, 1.08 (1.05–1.11) for stage 1 hypertension, and 1.20 (1.17–1.24) for stage 2 hypertension. Restricted cubic spline analysis showed an increase in CVD risk at SBP levels above ~130 mmHg. However, among individuals receiving antihypertensive medication, a U-shaped association was observed, with an increased risk also evident at SBP levels below ~130 mmHg. In individuals with non-proteinuric CKD without diabetes, higher SBP was positively associated with increased cardiovascular risk. However, a U-shaped association observed among individuals receiving antihypertensive treatment suggests that the relationship between BP and cardiovascular risk may be complex in this population, highlighting the potential importance of individualized clinical assessment.

**Figure:**
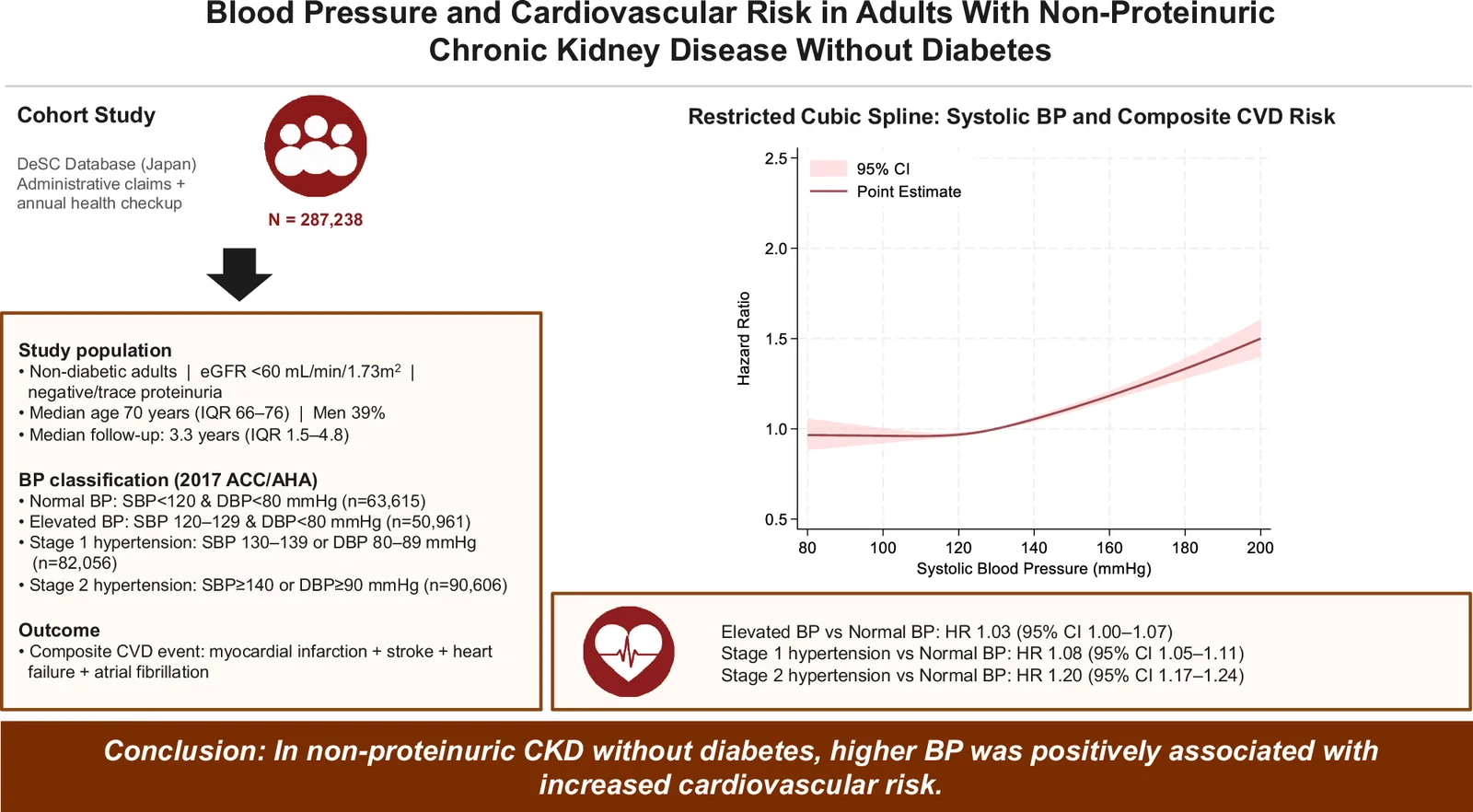

## Introduction

Chronic kidney disease (CKD) and hypertension are major determinants of cardiovascular disease (CVD) [1–3]. Current guidelines recognize both reduced estimated glomerular filtration rate (eGFR) and proteinuria (albuminuria) as key markers of CKD-related cardiovascular risk [4, 5]. However, a substantial proportion of individuals exhibit reduced eGFR in the absence of proteinuria, and this non-proteinuric CKD phenotype has become increasingly prevalent with population aging [6–9].

Despite its growing clinical importance, the role of blood pressure (BP) levels in CVD prevention and management in this population has not been fully established. Previous studies examining the association between BP and CVD have often evaluated CKD as a homogeneous condition, without distinguishing between proteinuric and non-proteinuric phenotypes [10–14]. In addition, the importance of intensive blood pressure control has been particularly emphasized in patients with diabetes and proteinuria (albuminuria), in whom cardiovascular risks are most pronounced [4, 12–14]. As a result, it remains unclear whether higher BP is associated with increased cardiovascular risk among adults with non-proteinuric CKD without diabetes. Furthermore, prior observational studies in CKD populations have reported inconsistent findings, with some demonstrating linear associations between BP and CVD outcomes while others showed J-shaped or U-shaped relationships [2, 15–17]. These discrepancies may reflect effect modification by clinical characteristics such as age and antihypertensive medication use, but investigation of these modifications in the non-proteinuric CKD population remains lacking.

Using a nationwide health checkup and claims database, we conducted a cohort study of adults with eGFR <60 mL/min/1.73 m^2^ in the absence of proteinuria and diabetes. We aimed to clarify the association between BP levels and major CVD events and examine potential effect modifications by key clinical characteristics.

Point of view

- **Clinical relevance**
  Findings from this study underscore the importance of BP management in adults with non-proteinuric CKD without diabetes and suggest that individualized clinical assessment may be warranted, particularly in those with low SBP receiving antihypertensive treatment.
- **Future direction**
  Further research is needed to better characterize the association between BP and cardiovascular risk among patients receiving antihypertensive treatment.
- **Consideration for the Asian population**
  Although our findings suggest the importance of BP management and individualized clinical assessment in adults with non-proteinuric CKD without diabetes, CKD etiology and clinical characteristics vary across Asia, warranting further studies to assess the generalizability of the present findings.

## Methods

### Data source

We analyzed data from the DeSC database (DeSC Healthcare Inc., Tokyo, Japan), a large-scale administrative claims and health checkup database in Japan. This database comprises data from three types of health insurance schemes in Japan: (1) the National Health Insurance (covering self-employed individuals, retired employees, and their dependents); (2) association/union-administered health insurance for employees of relatively large companies; and (3) the Advanced Elderly Medical Service System (covering individuals aged 75 years or older) [18, 19]. The database stores anonymized individual-level administrative claims data from both inpatient and outpatient settings. Individual participants can be longitudinally followed throughout the study period, including across multiple healthcare institutions. Disease diagnostic information is recorded in accordance with the International Classification of Diseases, 10th Revision (ICD-10). In addition, the database includes annual health checkup data, comprising blood pressure measurements, body mass index (BMI), laboratory test results, and self-reported lifestyle information obtained through standardized questionnaires (e.g., cigarette smoking and alcohol consumption).

### Ethics statement

We conducted this study in accordance with the Declaration of Helsinki. The Ethics Committee of the University of Tokyo approved this study (approval number: 2021010NI). The requirement for informed consent was waived because all data in this data set were anonymized and de-identified.

### Study population

The flowchart of the study participant selection is shown in Supplementary Fig. 1. We identified 508,168 individuals who underwent health checkups and met the following criteria between April 2014 and August 2024: (1) an eGFR <60 mL/min/1.73 m^2^, (2) negative or trace proteinuria by dipstick urinalysis, and (3) no history of diabetes (defined by ICD-10 codes E10–E14). From this population, we excluded individuals who met one or more of the following criteria: a prior history of CVD (*n* = 124,395), a history of kidney replacement therapy (*n* = 116), missing data on cigarette smoking (*n* = 115,470), or missing data on alcohol consumption (*n* = 142,401). Consequently, a total of 287,238 individuals were included in the final analysis.

### Data collection and definitions

Baseline characteristics, including age, sex, BMI, BP, and laboratory parameters (hemoglobin A1c [HbA1c], lipid profiles, eGFR, and urinalysis), were obtained from health checkup data. eGFR was calculated using the following equation: eGFR = 194 × (serum creatinine)^−1.094^ × (age)^−0.287^, multiplied by 0.739 for women [20]. This equation was developed to account for the lower serum creatinine levels observed in Japanese individuals. It tends to yield lower eGFR estimates than the Chronic Kidney Disease Epidemiology Collaboration (CKD-EPI) equation in Japanese populations. Proteinuria was assessed using dipstick urinalysis; negative and trace results were defined as non-proteinuric. BP was measured using a standard sphygmomanometer. In accordance with the 2017 American College of Cardiology (ACC)/American Heart Association (AHA) guidelines, participants were categorized into four groups based on baseline BP levels: Normal BP (systolic BP [SBP] < 120 mmHg and diastolic BP [DBP] < 80 mmHg); Elevated BP (SBP 120–129 mmHg and DBP < 80 mmHg); Stage 1 hypertension (SBP 130–139 mmHg or DBP 80–89 mmHg); and Stage 2 hypertension (SBP ≥ 140 mmHg or DBP ≥ 90 mmHg) [21]. Information on lifestyle factors (smoking status and alcohol consumption) was collected via self-reported questionnaires. From the administrative claims data, we identified antihypertensive medication use based on prescription records within the six months before the initial health checkup, using the World Health Organization Anatomical Therapeutic Chemical (WHO-ATC) classification system (ATC code: C02, C03, C04, C07, C08, and C09). Dyslipidemia was defined as the presence of any of the following: a low-density lipoprotein cholesterol level of ≥140 mg/dL, a high-density lipoprotein cholesterol level of <40 mg/dL, a triglyceride level of ≥150 mg/dL, or the use of lipid-lowering medications (ATC code: C10).

### Outcomes

The primary outcome was a composite of CVD events, defined as myocardial infarction (MI) (ICD-10 codes: I210–I214 and I219), stroke (ICD-10 codes: I630, I631–I636, I638, I639, I600–I611, I613–I616, I619, I629, and G459), heart failure (HF) (ICD-10 codes: I500, I501, I509, and I110), or atrial fibrillation (AF) (ICD-10 code: I480–I484, and I489). Secondary outcomes included the individual components of the composite outcome. Data for each endpoint were collected between April 2014 and August 2024. Participants were followed from the date of the initial health checkup (i.e., blood pressure measurement) until the occurrence of the outcome, discontinuation of insurance coverage (e.g., change of insurance, relocation, or death), or the end of the study period (August 2024), whichever occurred first.

### Statistical analysis

We performed Cox proportional hazards regression analyses to estimate hazard ratios (HRs) and 95% confidence intervals (CIs) for the incidence of composite and individual CVD events. We constructed three models to adjust for potential confounders: Model 1: Unadjusted. Model 2: Adjusted for age and sex. Model 3: Adjusted for age, sex, BMI, antihypertensive medication use, HbA1c, dyslipidemia, cigarette smoking, alcohol consumption, eGFR, and proteinuria status (negative vs. trace). We analyzed BP both as a continuous variable (per 10 mmHg increase in SBP and DBP) and as a categorical variable (according to the BP categories defined by the 2017 ACC/AHA guidelines). The Schoenfeld residual plots indicated no violation of the proportional hazards assumption for the primary endpoint (Supplementary Fig. 2). Furthermore, to visualize the non-linear association between SBP/DBP and the risk of CVD, we employed restricted cubic spline models with 4 knots (5th, 35th, 65th, and 95th percentiles). An SBP of 130 mmHg and a DBP of 80 mmHg were set as the reference values. Non-linearity was assessed using a likelihood ratio test comparing a restricted cubic spline model with a model including SBP as a linear term.

Subgroup analyses were performed stratified by age (≥70 vs. <70 years), sex (men vs. women), proteinuria status (negative vs. trace), eGFR (≥54.9 vs. <54.9 mL/min/1.73 m^2^ [median value]), and antihypertensive medication use (with vs. without).

Eight sensitivity analyses were performed to confirm the robustness of the primary analysis results. First, we reran the analysis using Fine and Gray competing risk regression to model the subdistribution hazard while accounting for the competing risk of mortality. Second, we performed multiple imputations using chained equations to impute missing data on cigarette smoking and alcohol consumption. Missing values were assumed to be missing at random, and 20 imputations were generated. Third, to minimize the potential influence of reverse causation, we applied a 1-year induction period and excluded participants who experienced CVD events within the first year of follow-up. A similar analysis was additionally conducted as a post hoc subgroup analysis according to antihypertensive medication use. Fourth, incident HF was redefined as having at least two recorded diagnoses within a 3-month period, rather than a single recorded diagnosis. Fifth, we redefined diabetes status as HbA1c ≥ 6.5% or use of antidiabetic medications and repeated the primary analyses among individuals who did not meet these criteria. Sixth, we redefined the composite outcome to consist of MI, stroke, and HF, excluding AF. Seventh, to address potential misclassification of BP levels, we calculated the average of BP values obtained at the initial health checkup and one year later, and used the averaged BP value as the exposure variable to examine its association with the risk of composite CVD events. Eighth, given that CKD status was defined based on a single health checkup in the primary analysis, we restricted the study population to participants who met both criteria at both the initial health checkup and the subsequent health checkup one year later: eGFR <60 mL/min/1.73 m^2^ and negative or trace proteinuria by dipstick urinalysis.

As an additional analysis, we extended the cohort to include participants with positive proteinuria (≥1+ on dipstick urinalysis). We compared the association between SBP and the risk of composite CVD events across proteinuric and non-proteinuric CKD subgroups.

All statistical analyses were performed using Stata v19 software (StataCorp, College Station, TX, USA). A two-sided *P*-value < 0.05 was considered statistically significant.

## Results

### Baseline characteristics

The baseline characteristics of the 287,238 study participants are summarized in Table 1. The median age was 70 years (IQR 66–76), and 39.0% were men. The median eGFR was 54.9 mL/min/1.73 m^2^, and 11.0% of participants were categorized as having trace proteinuria. The prevalence of antihypertensive medication use was 37.9%. Supplementary Table 1 presents the baseline characteristics stratified by BP categories according to the 2017 ACC/AHA guidelines. The distribution was as follows: normal BP (*n* = 63,615), elevated BP (*n* = 50,961), stage 1 hypertension (*n* = 82,056), and stage 2 hypertension (*n* = 90,606). Participants with higher BP stages were more likely to be men, have a higher BMI, and have a higher prevalence of dyslipidemia and alcohol consumption.

**Table 1:** Baseline characteristics

	Total (*n* = 287,238)
Age, years	70 (66–76)
Men, *n* (%)	112,023 (39.0)
BMI, kg/m^2^	22.9 (20.9–25.0)
SBP, mmHg	130 (120–142)
DBP, mmHg	76 (69–83)
Antihypertensive medication, *n* (%)	108,901 (37.9)
Dyslipidemia, *n* (%)	188,667 (65.7)
Cigarette smoking, *n* (%)	24,946 (8.7)
Alcohol consumption, *n* (%)	56,929 (19.8)
HbA1c, %	5.6 (5.4–5.8)
LDL-C, mg/dL	126 (106–147)
HDL-C, mg/dL	62 (51–74)
Triglycerides, mg/dL	103 (75–144)
eGFR, ml/min per 1.73 m^2^	54.9 (50.8–57.7)
*Proteinuria, n (%)*	
Negative	255,549 (89.0)
Trace	31,689 (11.0)

Data are reported as medians (interquartile range) or numbers (percentage), where appropriate

*BMI* body mass index, *SBP* systolic blood pressure, *DBP* diastolic blood pressure, *LDL-C* low-density lipoprotein cholesterol, *HDL-C* high-density lipoprotein cholesterol, *eGFR* estimated glomerular filtration rate

### Association between blood pressure and cardiovascular events

**Fig. 1:**
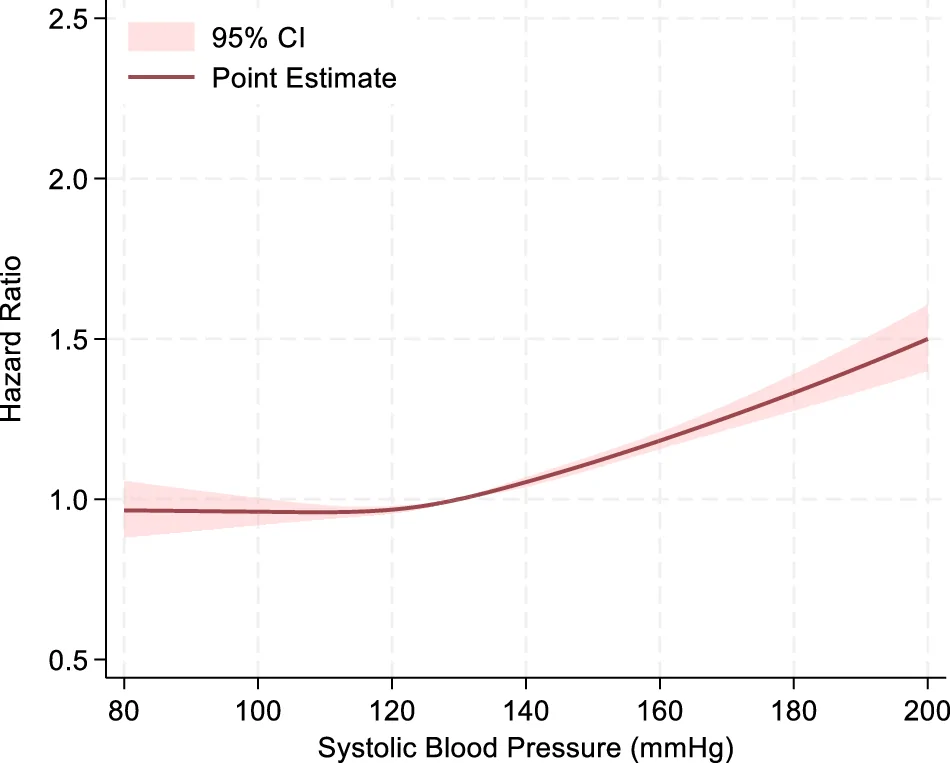
Hazard ratios for the development of composite cardiovascular disease with systolic blood pressure. The association between systolic blood pressure (continuous variable) and the risk of composite cardiovascular disease events was evaluated using restricted cubic spline models with 4 knots. The solid line represents the multivariable-adjusted hazard ratio, and the shaded area indicates the 95% confidence interval. The reference value was set at 130 mmHg. The model was adjusted for age, sex, body mass index, antihypertensive medication use, hemoglobin A1c, dyslipidemia, cigarette smoking, alcohol consumption, estimated glomerular filtration rate, and proteinuria status. *P* for non-linearity was <0.01

During the median follow-up period of 3.3 years (IQR 1.5–4.8), 40,548 composite CVD events were recorded. In the continuous variable analysis (Supplementary Table 2), both SBP and DBP were significantly associated with the risk of composite CVD events. In Model 3, the HR was 1.05 (1.04–1.05) per 10 mmHg increase in SBP and 1.06 (1.05–1.07) per 10 mmHg increase in DBP. Restricted cubic spline analyses visually confirmed a positive association between SBP (Fig. 1) and DBP (Supplementary Fig. 3) and the risk of composite CVD events, with the risk increasing monotonically above 130 mmHg for SBP and above 80 mmHg for DBP. In the categorical analysis (Table 2), a graded association was observed between higher BP categories and an increased risk of composite CVD events. In the fully adjusted model (Model 3), compared with the normal BP group, the HRs (95% CIs) for elevated BP, stage 1 hypertension, and stage 2 hypertension were 1.03 (1.00–1.07), 1.08 (1.05–1.11), and 1.20 (1.17–1.24), respectively.

**Table 2:** Blood pressure category according to the 2017 ACC/AHA BP guidelines and composite cardiovascular events

	Normal BP (*n* = 63,615)	Elevated BP (*n* = 50,961)	Stage 1 hypertension (*n* = 82,056)	Stage 2 hypertension (*n* = 90,606)
Number of events	6856	7031	11,631	15,030
Crude IR (95% CI)	346.9 (338.6–355.1)	462.9 (452.1–473.6)	479.9 (471.2–488.6)	582.7 (573.4–591.9)
Adjusted IR (95% CI)	439.4 (428.9–449.9)	453.3 (442.9–463.8)	473.5 (464.9–482.0)	526.3 (517.9–534.8)
Adjusted IRD (95% CI)	0 [Reference]	13.9 (−0.9–28.7)	34.0 (20.4–47.6)	86.9 (73.2–100.6)
Hazard ratio (95% CI)				
Model 1	1 [Reference]	1.33 (1.29–1.38)	1.38 (1.34–1.42)	1.68 (1.63–1.73)
Model 2	1 [Reference]	1.07 (1.03–1.10)	1.13 (1.09–1.16)	1.26 (1.23–1.30)
Model 3	1 [Reference]	1.03 (1.00–1.07)	1.08 (1.05–1.11)	1.20 (1.17–1.24)

IR and IRD are presented per 10,000 person-years. Crude and adjusted IR were estimated using Poisson regression models; adjusted IR were obtained using marginal standardization. Adjusted IR and IRD were estimated after adjustment for age, sex, body mass index, antihypertensive medication use, hemoglobin A1c, dyslipidemia, cigarette smoking, alcohol consumption, estimated glomerular filtration rate, and proteinuria status. Hazard ratios were estimated using Cox proportional hazards regression models. Model 1 is unadjusted. Model 2 includes adjustment for age and sex. Model 3 includes adjustment for age, sex, body mass index, antihypertensive medication use, hemoglobin A1c, dyslipidemia, cigarette smoking, alcohol consumption, estimated glomerular filtration rate, and proteinuria status

*95% CI* 95% confidence interval, *ACC* American College of Cardiology, *AHA* American Heart Association, *BMI* body mass index, *BP* blood pressure, *IR* incidence rate, *IRD* incidence rate difference

### Individual cardiovascular outcomes

**Fig. 2:**
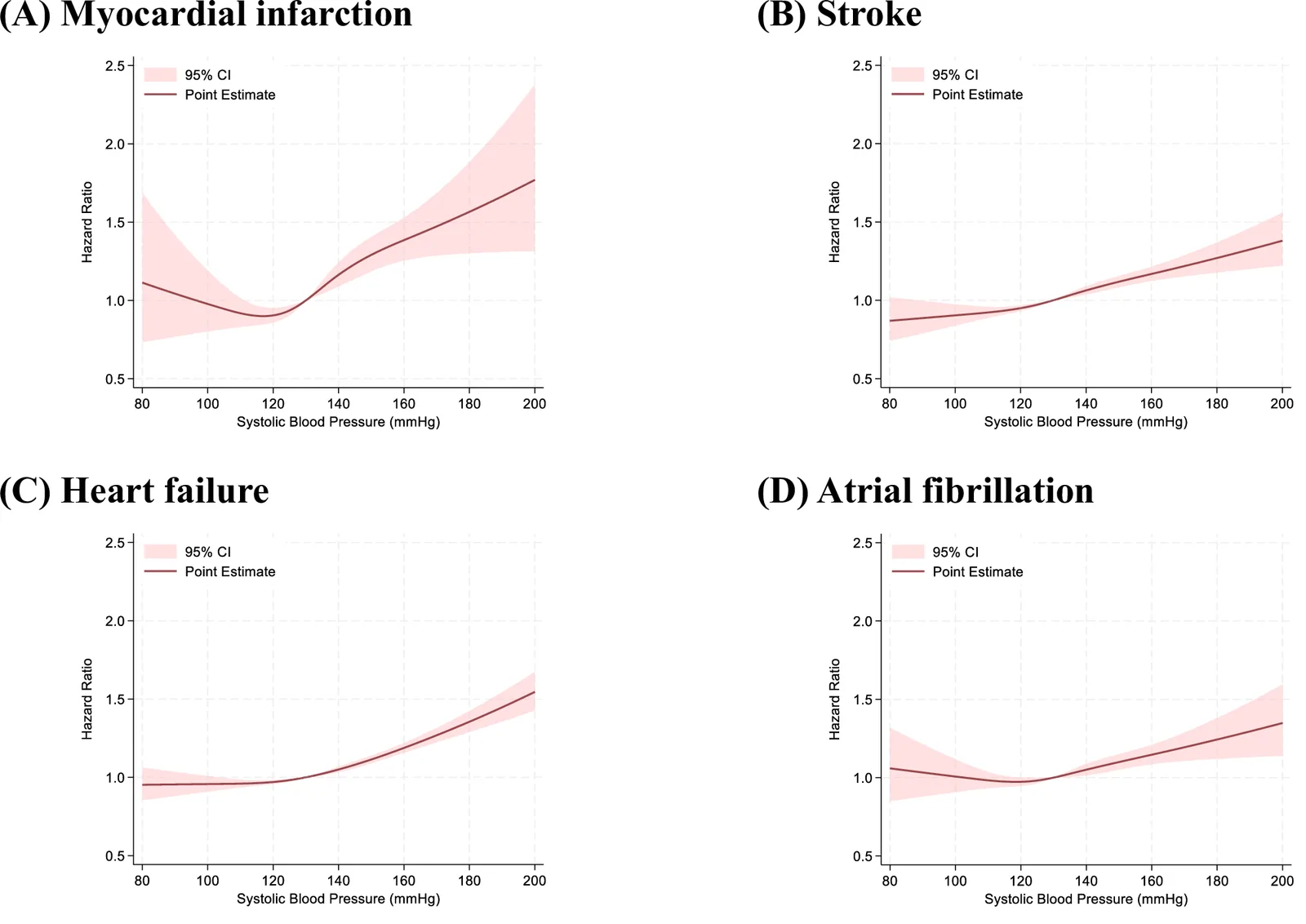
Hazard ratios for the development of each cardiovascular disease with systolic blood pressure. Restricted cubic spline plots showing the association between systolic blood pressure and the risk of **A** myocardial infarction, **B** stroke, **C** heart failure, and **D** atrial fibrillation. The solid lines and shaded areas represent the hazard ratios and 95% confidence intervals, respectively. The model was adjusted for age, sex, body mass index, antihypertensive medication use, hemoglobin A1c, dyslipidemia, cigarette smoking, alcohol consumption, estimated glomerular filtration rate, and proteinuria status. *P* for non-linearity was 0.03 for myocardial infarction, 0.42 for stroke, <0.01 for heart failure, and 0.10 for atrial fibrillation

The number of events for each individual component was as follows: MI, 1952; stroke, 13,329; HF, 28,972; and AF, 6952. The associations between SBP and individual CVD outcomes (MI, stroke, HF, and AF) are shown in Fig. 2. Higher SBP was associated with an increased risk of all individual outcomes. Specifically, the risk of MI showed a steep increase with rising SBP. Similar trends were observed for DBP (Supplementary Fig. 3).

### Subgroup analyses

**Fig. 3:**
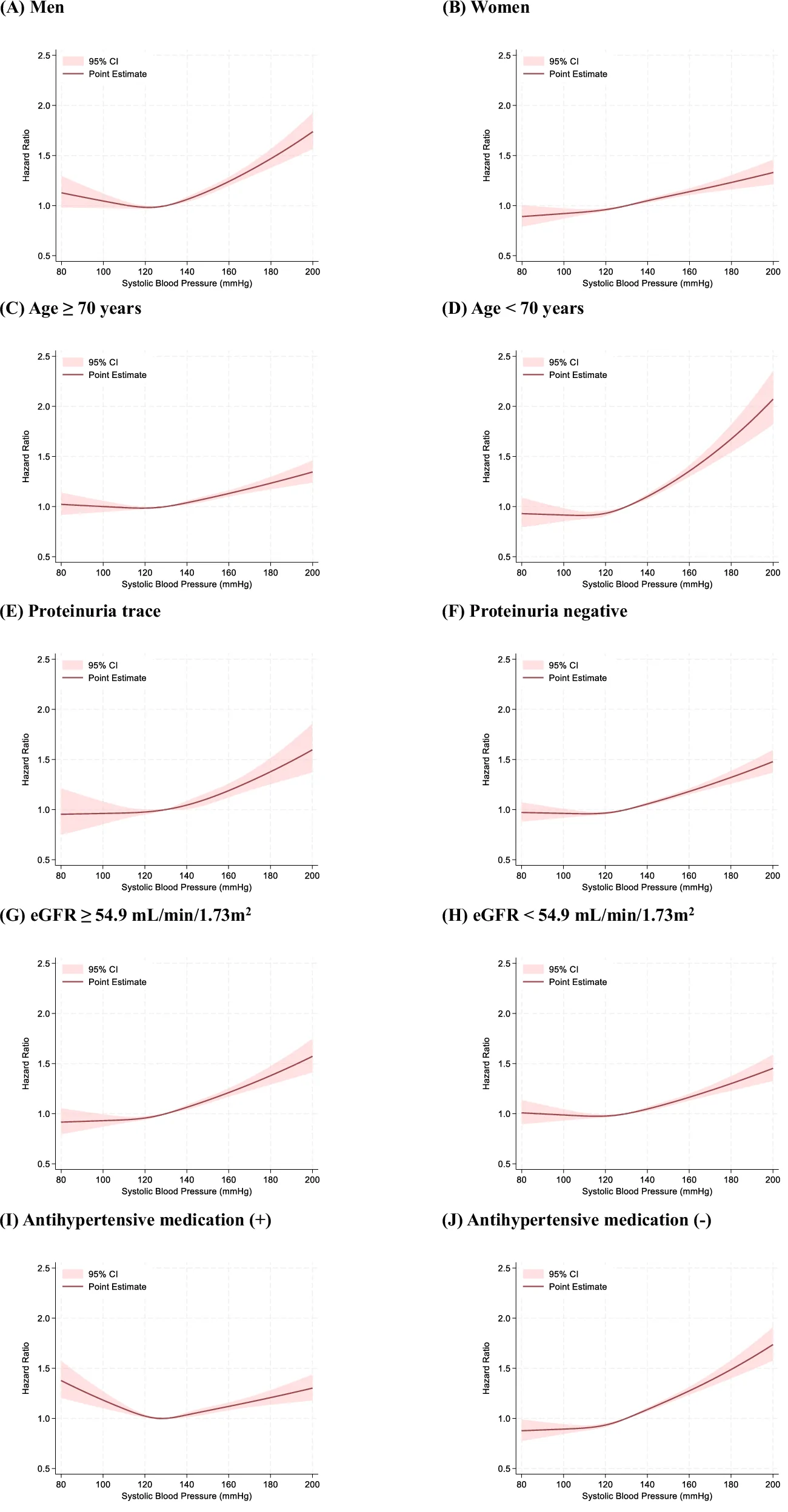
Subgroup analysis. Restricted cubic spline analyses stratified by clinical characteristics: **A** Men, **B** Women, **C** age ≥70 years, **D** age <70 years, **E** trace proteinuria, **F** negative proteinuria, **G** eGFR ≥54.9 mL/min/1.73 m^2^, **H** eGFR <54.9 mL/min/1.73 m^2^, **I** with antihypertensive medication, and **J** without antihypertensive medication

The results of subgroup analyses for the association between SBP and composite CVD events are presented in Fig. 3. The positive association between SBP and CVD risk was generally consistent across subgroups stratified by sex (men vs. women) (Fig. 3A, B), age (≥70 vs. < 70 years) (Fig. 3C, D), proteinuria status (trace vs. negative) (Fig. 3E, F), and eGFR levels (≥54.9 vs. <54.9 mL/min/1.73 m^2^) (Fig. 3G, H). However, a U-shaped association was observed in individuals taking antihypertensive medication, where the risk of CVD events increased with both higher and lower BP (Fig. 3I), whereas the positive association between SBP and CVD risk was observed in individuals not taking antihypertensive medication (Fig. 3J). *P* values for interactions between SBP and sex, age, proteinuria, eGFR, and antihypertensive medication were <0.01, <0.01, 0.73, 0.01, and <0.01, respectively. Subgroup analyses examining the association between BP categories based on the 2017 ACC/AHA guidelines and risk of composite CVD events are shown in Supplementary Table 3. Regardless of sex, age, proteinuria, or eGFR status, individuals with stage 2 hypertension consistently had a higher risk of CVD than those with normal BP. In contrast, stage 2 hypertension was not significantly associated with an increased CVD risk compared with normal BP among individuals taking antihypertensive medications (HR 1.01, 95% CI 0.96–1.05) (Supplementary Table 3).

### Sensitivity analyses

First, the positive association between SBP and an increased risk of composite CVD events remained unchanged in the competing risk model (Supplementary Fig. 4). Second, after performing multiple imputation for missing data on cigarette smoking and alcohol consumption, we analyzed 383,717 individuals. Baseline characteristics stratified by missing status of covariates are presented in Supplementary Table 4. The positive association between SBP and increased risk of composite CVD events was consistent with that observed in the primary analyses (Supplementary Fig. 5). In analyses using BP categories, compared with the normal BP group, the HRs (95% CIs) for elevated BP, stage 1 hypertension, and stage 2 hypertension were 1.02 (0.99–1.05), 1.04 (1.02–1.07), and 1.16 (1.13–1.19), respectively (Supplementary Table 5). Third, the primary findings were unchanged in this sensitivity analysis, which included 224,973 participants after applying an induction period of 1 year (Supplementary Fig. 6). Consistent with the primary analysis, a U-shaped association was also observed among individuals taking antihypertensive medication (Supplementary Fig. 7). Fourth, incident HF was redefined as requiring at least two ICD-10 diagnosis codes recorded within a 3-month period. Higher SBP was associated with an increased risk of HF under this definition (Supplementary Fig. 8). Fifth, in a sensitivity analysis using an alternative definition of diabetes (HbA1c ≥ 6.5% or use of antidiabetic medications), 320,186 individuals were included. The association between SBP and composite CVD risk remained consistent with that observed in the primary analysis (Supplementary Fig. 9). Sixth, in a sensitivity analysis redefining the composite outcome as MI, stroke, and HF, the association between higher SBP and the composite CVD outcome remained unchanged (Supplementary Fig. 10). Seventh, among the 156,027 participants with BP measurements available one year later, the positive association between higher SBP, defined using the average of BP values obtained at the initial health checkup and one year later, and the risk of composite CVD events was similar to the findings of the primary analysis (Supplementary Fig. 11). Eighth, among the 115,939 participants who met both the eGFR and proteinuria criteria at the initial health checkup and at the subsequent health checkup one year later, higher SBP was associated with a higher risk of composite CVD events (Supplementary Fig. 12).

In an additional analysis extending the cohort to include 22,325 participants with proteinuria, the positive association between SBP and composite CVD risk was also observed among individuals with proteinuria (Supplementary Fig. 13). The magnitude of the association with higher SBP was more pronounced in the proteinuric subgroup, particularly at higher SBP levels (*P* for interaction = 0.01).

## Discussion

In this large-scale, nationwide cohort study utilizing an integrated database of health checkups and administrative claims, we analyzed 287,238 adults with reduced eGFR (<60 mL/min/1.73 m^2^) but negative or trace proteinuria without diabetes. We observed a graded association between higher SBP and the risk of subsequent composite CVD events. Subgroup analyses showed that this positive association was generally consistent across age and sex, whereas a U-shaped association was observed among individuals receiving antihypertensive medication. These findings underscore the importance of BP monitoring as part of CVD risk assessment in adults with non-proteinuric CKD. Further research is warranted to better characterize the relationship between BP and cardiovascular risk among patients receiving antihypertensive treatment.

### Comparison with previous studies

Numerous previous studies have investigated BP targets in patients with CKD. However, most have treated CKD as a homogeneous entity, potentially obscuring important differences across various clinical characteristics, including proteinuria status and eGFR levels [10, 22–24]. In particular, data specifically addressing the BP–CVD risk relationship in non-proteinuric CKD without diabetes have been relatively limited and often derived from subgroup analyses with modest sample sizes. Our study addresses this gap by leveraging a large cohort of nearly 300,000 individuals with non-proteinuric renal dysfunction, enabling a focused investigation of this specific population. Our finding of an increase in cardiovascular risk with rising SBP aligns with general population studies but adds critical granularity for this specific phenotype [25]. While several previous reports in CKD have suggested a J- or U-shaped curve [26, 27], our results in a broader cohort indicate that for most patients with mild-to-moderate renal dysfunction and no proteinuria, lower SBP is associated with better cardiovascular outcomes.

### Interpretation of findings and clinical implications

The present findings add to the growing body of evidence characterizing the association between BP and cardiovascular risk in adults with non-proteinuric CKD without diabetes. First, similar to the ACC/AHA criteria, the Japanese Society of Hypertension Guidelines for the Management of Elevated Blood Pressure and Hypertension 2025 (JSH2025) recommend that, in CKD without proteinuria, BP should be cautiously lowered to <130/80 mmHg, with 140/90 mmHg as an intermediate target, while giving consideration to adverse events such as worsening kidney function [28]. JSH2025 further notes that, particularly in older individuals with renal arteriosclerosis, careful consideration should be given to the potential for reduced organ perfusion associated with BP lowering. Although the guideline statement and our observational findings are not directly comparable, this caution regarding reduced organ perfusion during BP lowering may provide a clinically relevant framework for interpreting the U-shaped association observed among individuals receiving antihypertensive treatment in the present study.

Second, our additional analysis suggested that the association between higher SBP and CVD risk may be stronger among individuals with proteinuria than among those without proteinuria. Nevertheless, in the primary study population of adults with non-proteinuric CKD without diabetes, higher SBP was consistently associated with increased CVD risk. This finding suggests that reduced eGFR itself may identify a population with heightened vascular vulnerability, even in the absence of overt proteinuria.

Third, the association between BP and CVD risk observed across multiple subgroups in the present study has important clinical implications. The present study identified several patient characteristics, including sex and age, as significant modifiers of the association between elevated SBP and CVD risk. The stronger association between elevated SBP and CVD risk observed in men may be explained, at least in part, by a greater ambulatory BP burden that is not fully captured by office BP measurements. In a cohort study of adults with CKD stages 2–5, men had higher daytime and nighttime SBP than women on ambulatory BP monitoring, despite no sex differences in office BP [29]. The same study suggested that poorer ambulatory BP control in men may partly account for their higher risks of end-stage kidney disease and all-cause mortality. The stronger association between higher SBP and CVD risk in younger adults is consistent with findings from a large cohort study of veterans with CKD, which showed that the associations of higher SBP with incident coronary heart disease and stroke were strongest in younger adults and progressively attenuated with advancing age [30]. Our findings extend these prior observations by showing that sex and age may be important clinical factors in interpreting the association between elevated SBP and CVD risk among adults with non-proteinuric CKD without diabetes. Conversely, among those receiving antihypertensive treatment, we observed a U-shaped association, with cardiovascular risk increasing not only at higher SBP levels but also at lower levels below ~130 mmHg. This finding may reflect the complex clinical profile of treated individuals and does not provide a definitive answer as to whether lower BP itself exerts harmful effects. Several mechanisms may underlie the increased risk observed at lower SBP levels in the treated subgroup. Treated individuals with low SBP may include those with subclinical heart failure or reduced cardiac output. In some of these individuals, renin–angiotensin system inhibitors or beta-blockers may be prescribed primarily for cardiac indications, and low BP may reflect underlying cardiac dysfunction rather than successful BP lowering. The U-shaped association observed for MI may be consistent with this interpretation, because lower SBP in treated individuals may partly reflect an unfavorable hemodynamic profile, including lower DBP and impaired coronary perfusion [31–33]. Indeed, the Study of Heart and Renal Protection (SHARP) trial reported that the overall association between SBP and cardiovascular risk in CKD was U-shaped, whereas among participants with a low probability of subclinical CVD based on troponin-I levels, higher SBP was consistently associated with higher cardiovascular risk [16]. The treated subgroup may also include individuals with primary glomerular diseases, such as IgA nephropathy, in whom intensive BP lowering is pursued to maintain remission of proteinuria. In addition, the treatment burden associated with intensive BP control, including polypharmacy and adverse effects such as orthostatic hypotension, may attenuate or offset the potential cardiovascular benefits of lower BP in some individuals [26, 34–36].

### Strengths and limitations

The primary strength of this study is its large sample size and the use of a real-world dataset that combines health checkup data with administrative claims records. This allowed us to identify a large cohort of patients with non-proteinuric CKD without diabetes and conduct robust subgroup analyses. However, several limitations must be acknowledged. First, BP was measured at a single time point during a health checkup, which may not reflect usual BP due to variability or white-coat effects. The 2017 ACC/AHA guideline recommends that BP classification be based on the average of two or more readings obtained on at least two separate occasions [21]. This difference in BP assessment may have introduced exposure misclassification. Second, eGFR and proteinuria were assessed at a single time point during a health checkup. This single-occasion assessment may have introduced misclassification of CKD status, potentially biasing the observed associations. Third, proteinuria was assessed by dipstick, which is less sensitive than the albumin-to-creatinine ratio. Some non-proteinuric individuals might have had low-grade albuminuria. Fourth, given the observational nature of this study, residual confounding cannot be ruled out. In particular, the absence of data on cardiac function at baseline may have complicated the interpretation of the association between SBP and CVD risk among individuals receiving antihypertensive treatment. Fifth, the relatively short follow-up period of the present study may have introduced some uncertainty in the assessment of the association between BP levels and the risk of cardiovascular outcomes. Sixth, the definitions of cardiovascular outcomes relied solely on ICD-10 codes, and the positive and negative predictive values of ICD-10 codes alone have not been fully validated in Japanese claims databases, particularly for heart failure. Seventh, because the study population was restricted to individuals with available health checkup data, selection bias cannot be entirely ruled out. Further validation studies using independent datasets are warranted to confirm our findings. Finally, because our cohort consisted of relatively older Japanese adults, caution is warranted when generalizing these findings to CKD populations with different age distributions or ethnic backgrounds, in whom cardiovascular risk profiles may differ.

### Perspective of Asia

Our findings reinforce the clinical significance of BP control in adults with non-proteinuric CKD without diabetes. However, even within Asia, the spectrum of CKD, including its underlying etiology and clinical characteristics, varies across populations [37, 38]. Further studies are therefore warranted to confirm the generalizability of these findings across the region.

## Conclusions

In this large-scale cohort of adults with non-proteinuric CKD without diabetes, higher BP was associated with increased risk of major CVD events, including MI, stroke, HF, and AF. This association was observed across most key clinical subgroups. Although the present study does not provide a definitive answer as to whether lower BP itself may exert harmful effects, a U-shaped association was observed among individuals receiving antihypertensive treatment. These findings highlight the importance of BP management in adults with non-proteinuric CKD without diabetes, while also suggesting that individualized clinical assessment may be warranted, particularly in individuals receiving antihypertensive treatment who have low SBP. Further randomized trials are needed to clarify the cardiovascular effects of BP lowering in adults with non-proteinuric CKD without diabetes, because current trial evidence for this specific population remains limited.

## Supplementary information

Supplementary Tables

Supplementary Figures
