# Supplementary material for: Blood pressure and cardiovascular risk in adults with non-proteinuric chronic kidney disease without diabetes
Source: Hypertens Res. 2026 Sep 10;49(11):2999–3009. doi: 10.1038/s41440-026-02801-7 (PMC13736525; doi:10.1038/s41440-026-02801-7)

Supplementary Figure 1. Flow Chart

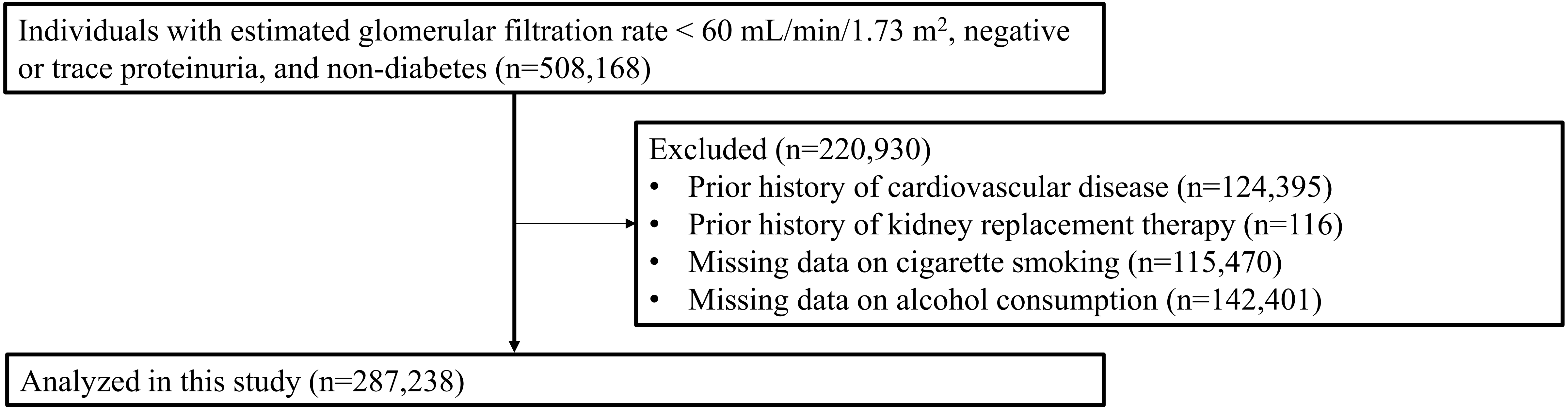

Supplementary Figure 2. Schoenfeld residual plots

(A) Systolic blood pressure

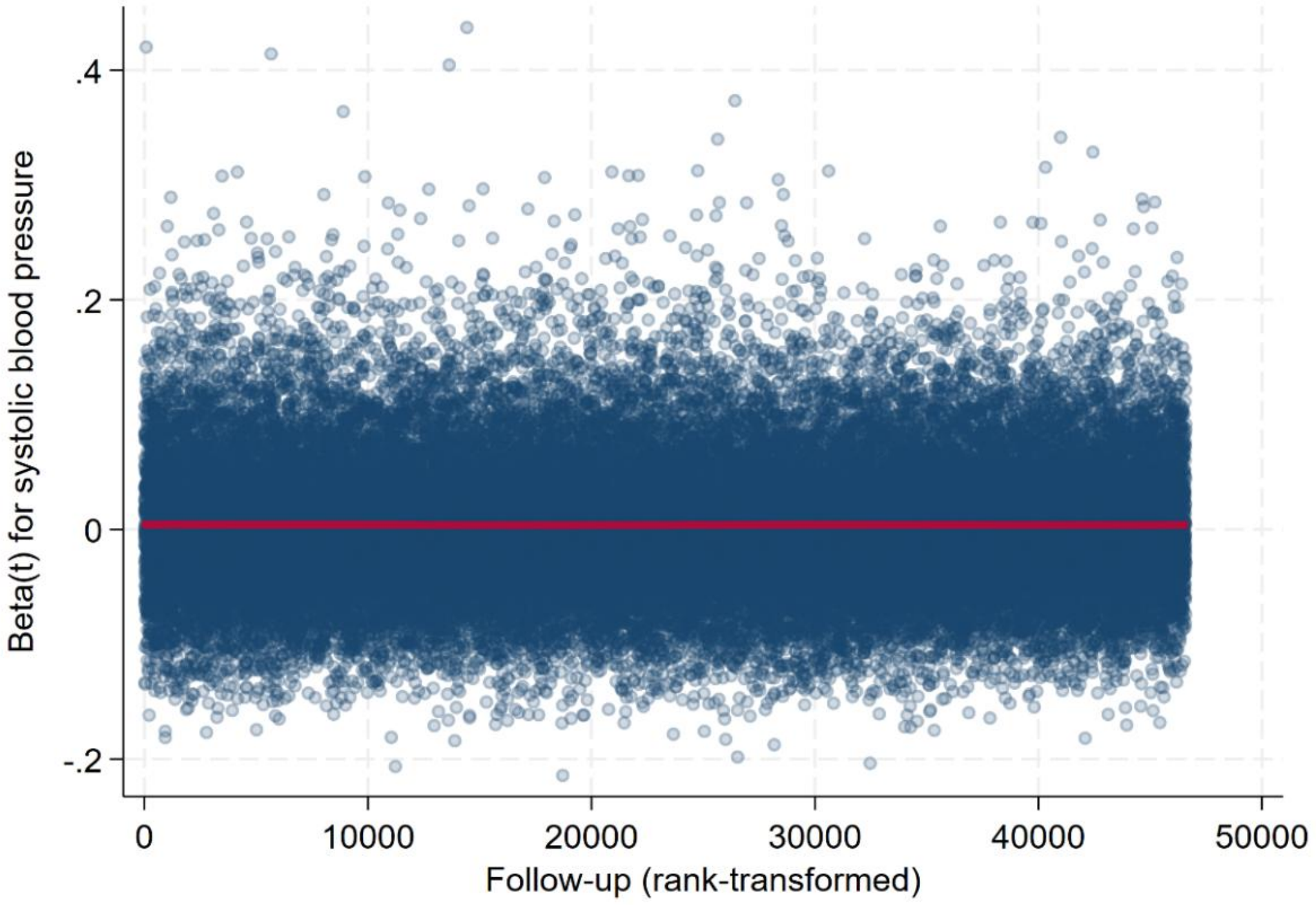

(B) Diastolic blood pressure

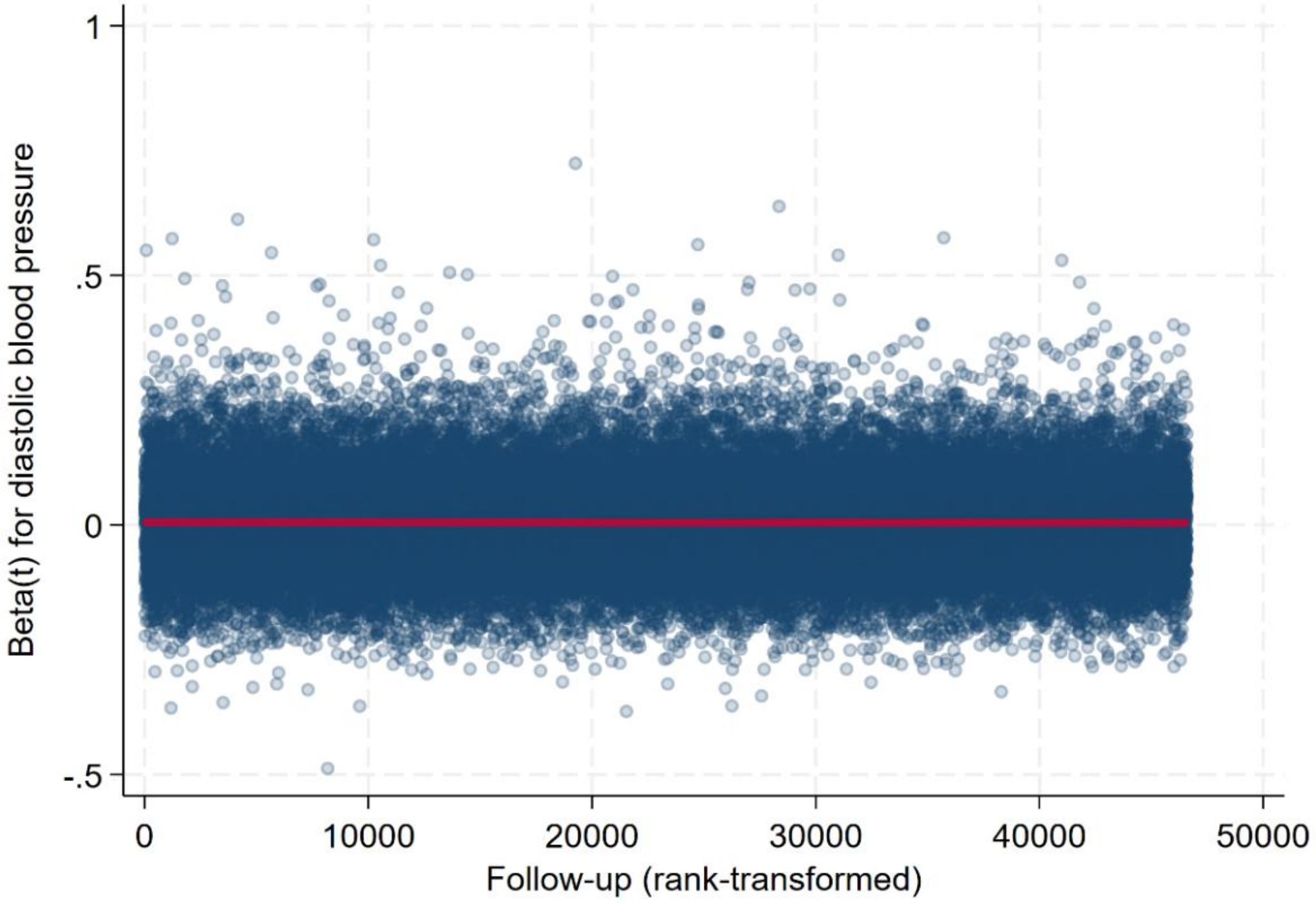

Supplementary Figure 3. Hazard ratios for the development of each cardiovascular disease with diastolic blood pressure

(A) Composite event

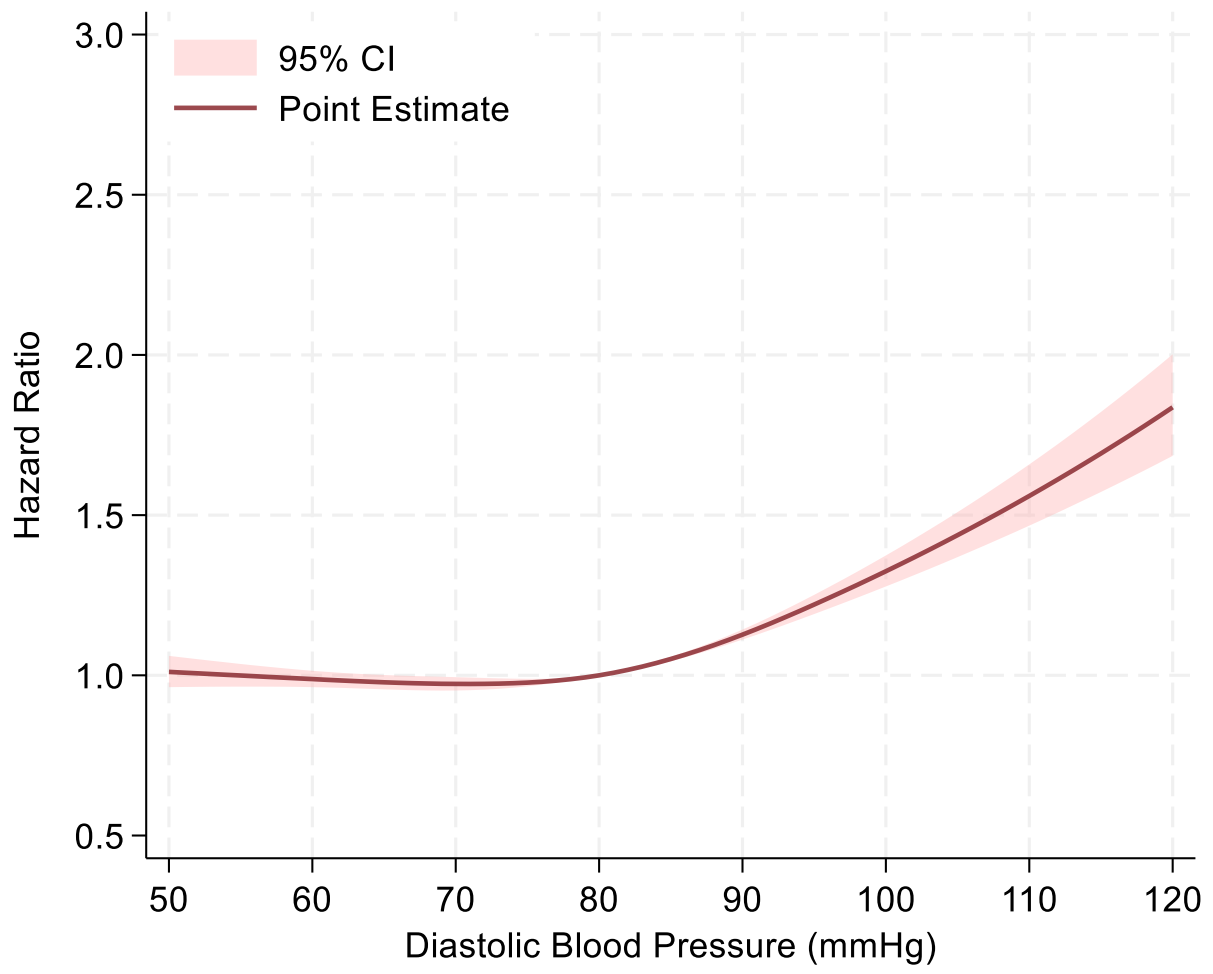

(B) Myocardial infarction

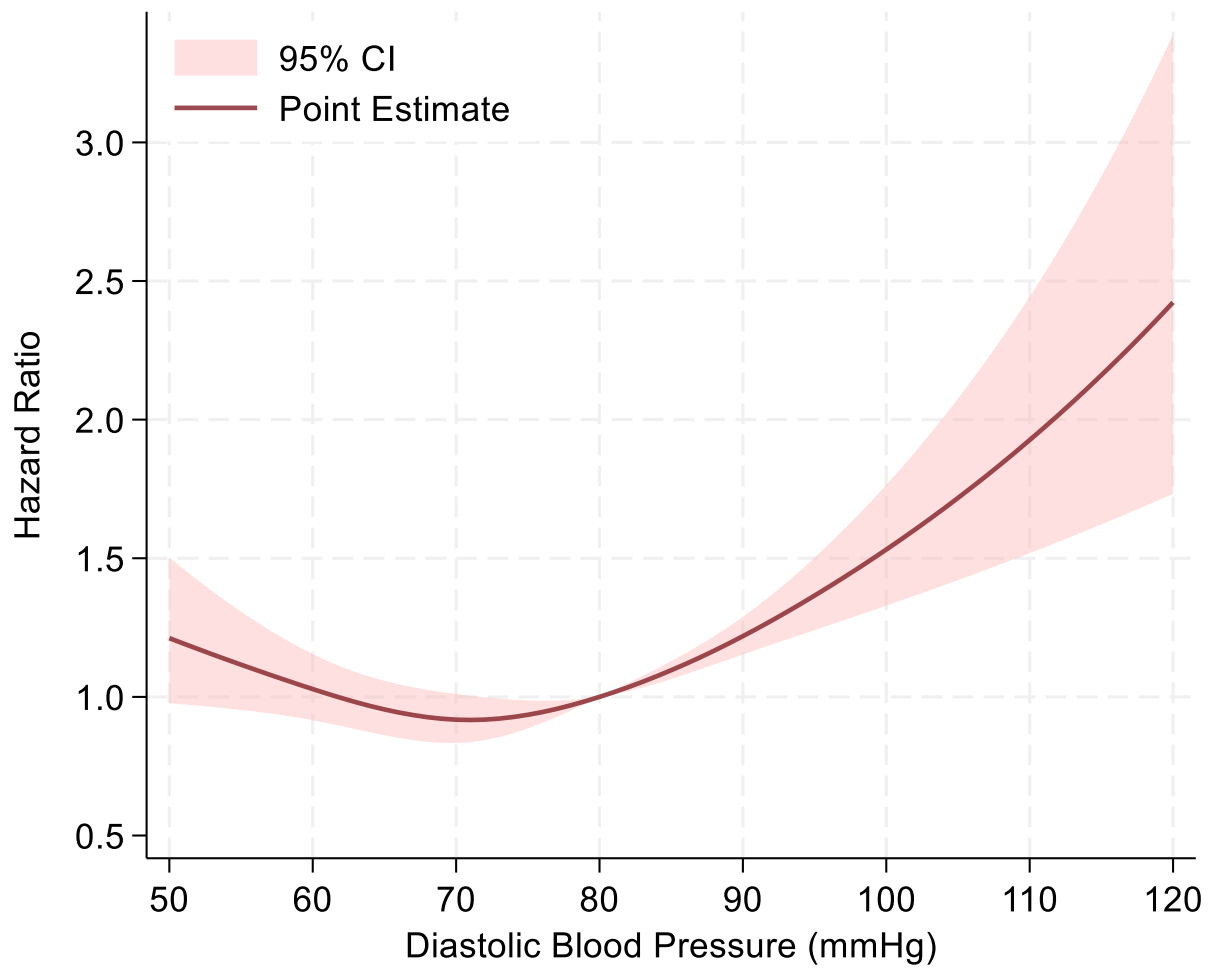

(C) Stroke

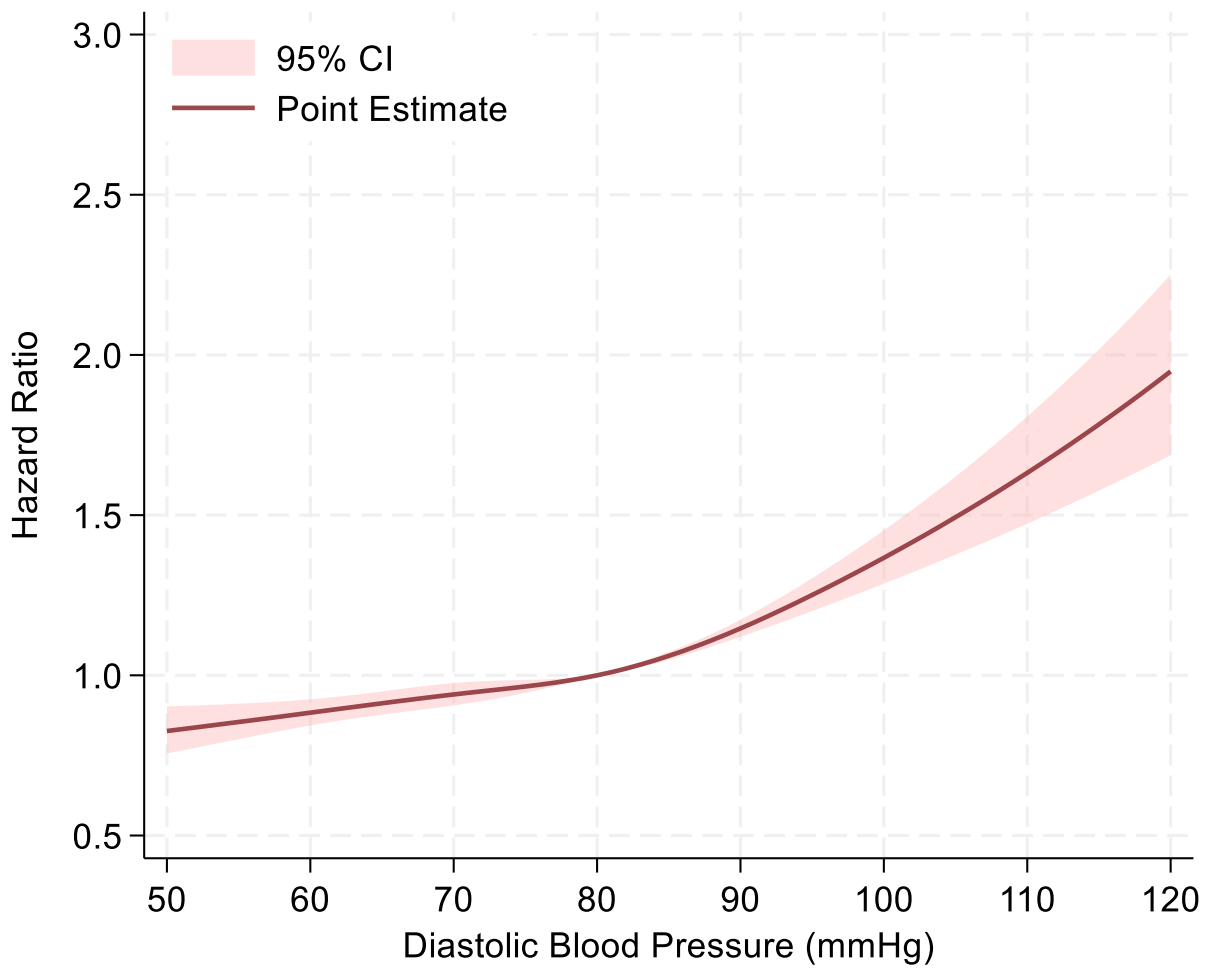

(D) Heart failure

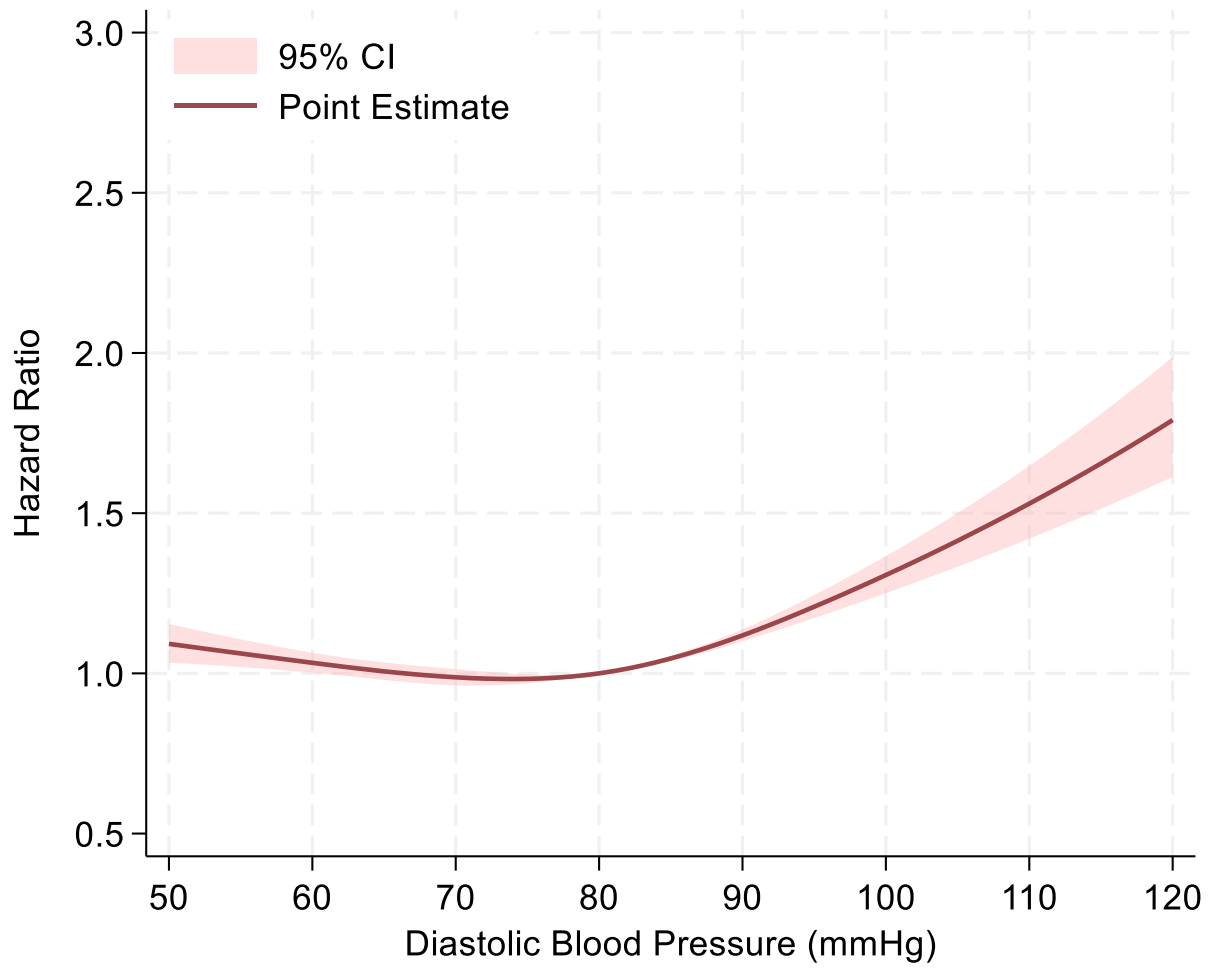

(E) Atrial fibrillation

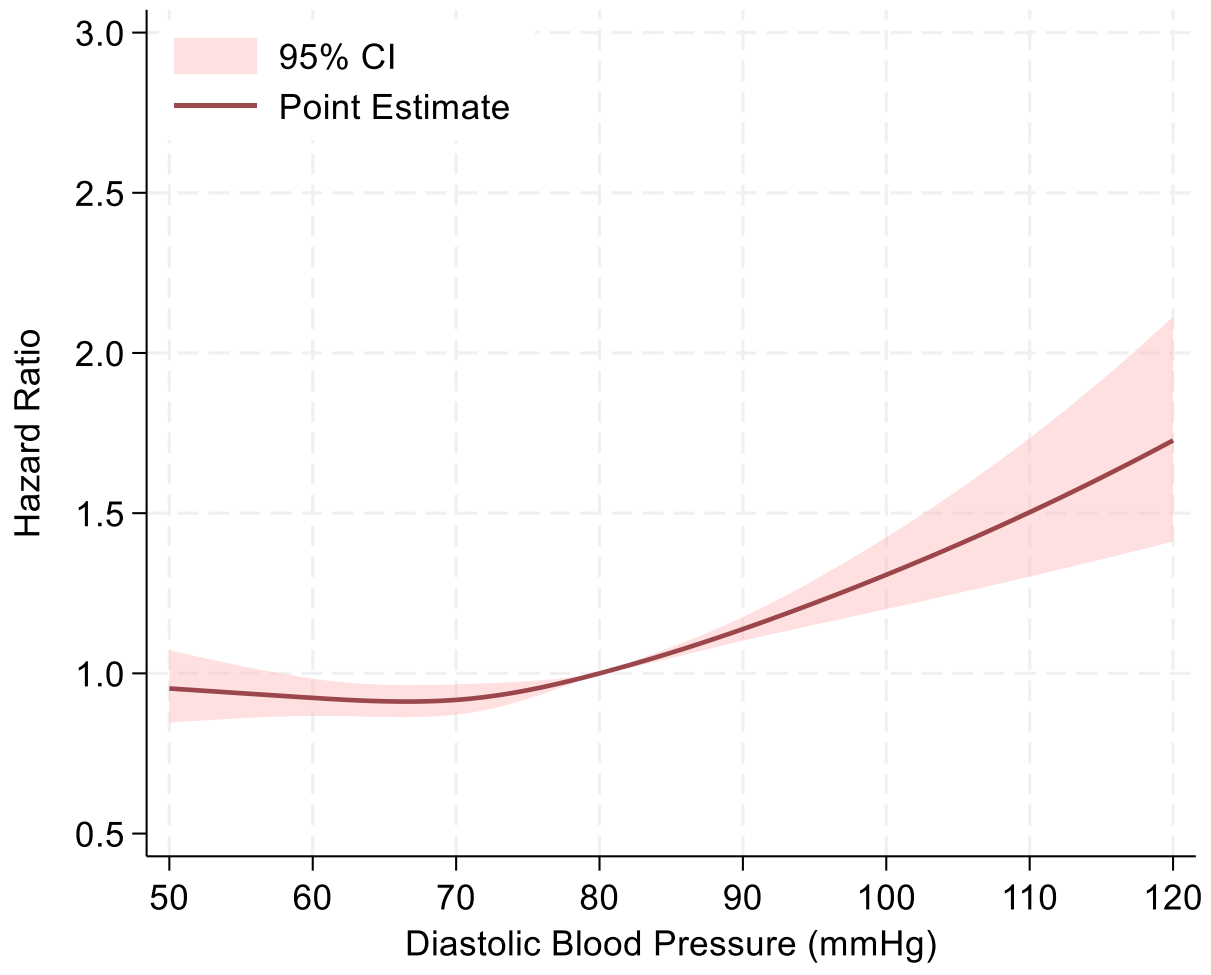

Supplementary Figure 4. Risk of developing composite cardiovascular disease according to systolic blood pressure in competing risk analysis

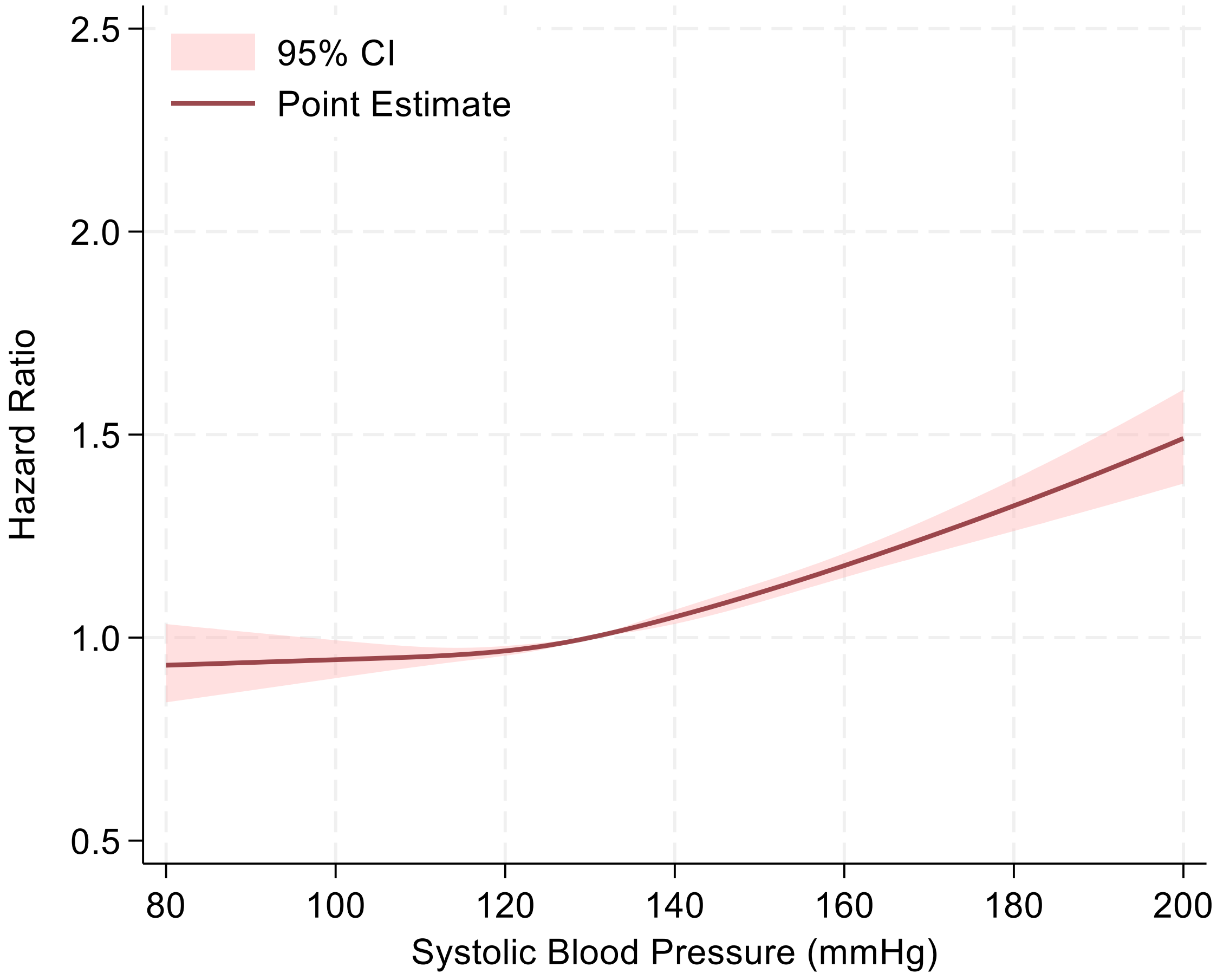

Supplementary Figure 5. Risk of developing composite cardiovascular disease according to systolic blood pressure in multiple imputations

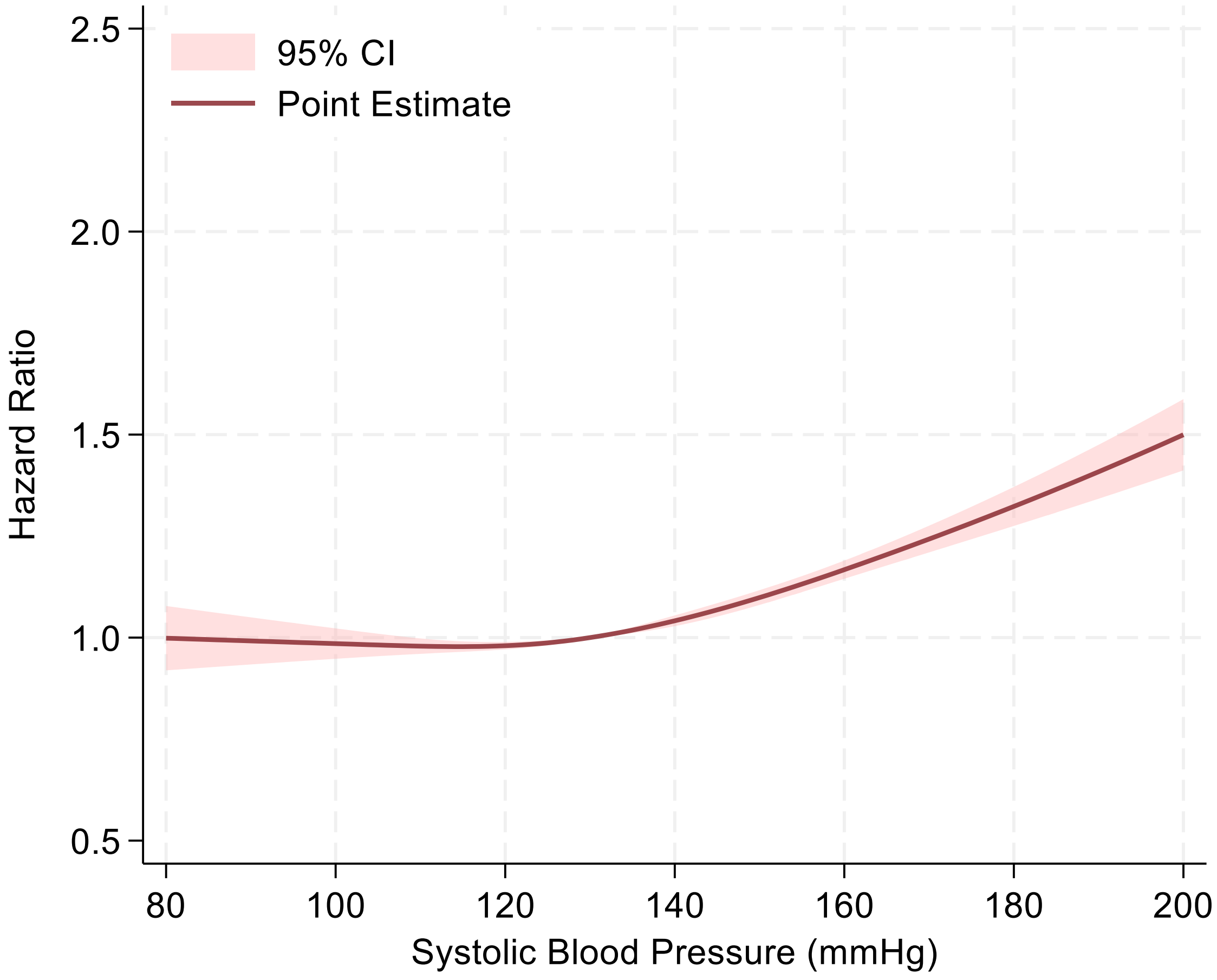

Supplementary Figure 6. Risk of developing composite cardiovascular disease according to systolic blood pressure (induction period of one year)

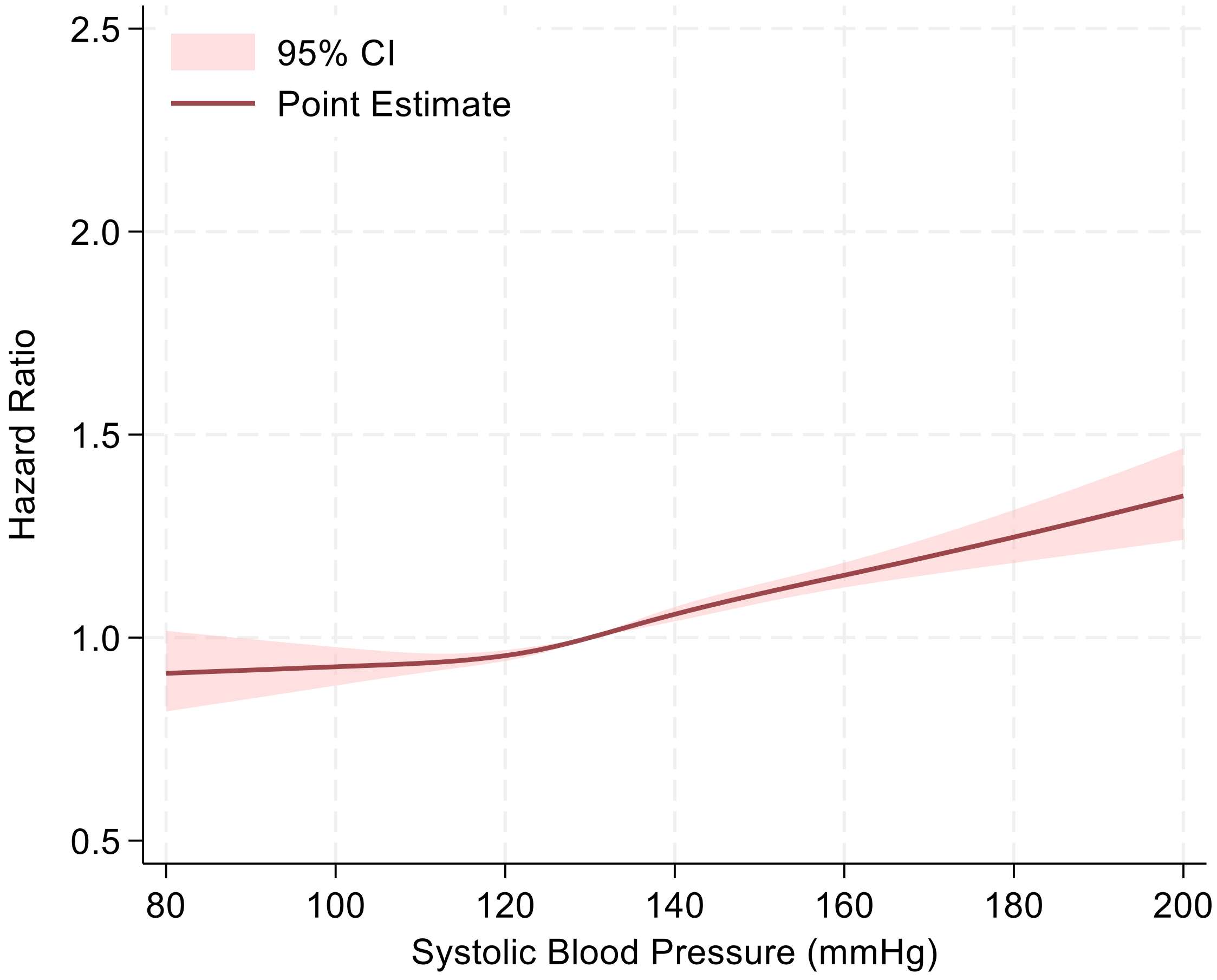

Supplementary Figure 7. Risk of developing composite cardiovascular disease according to systolic blood pressure with a 1-year induction period, stratified by antihypertensive medication use

(A) Antihypertensive medication (+)

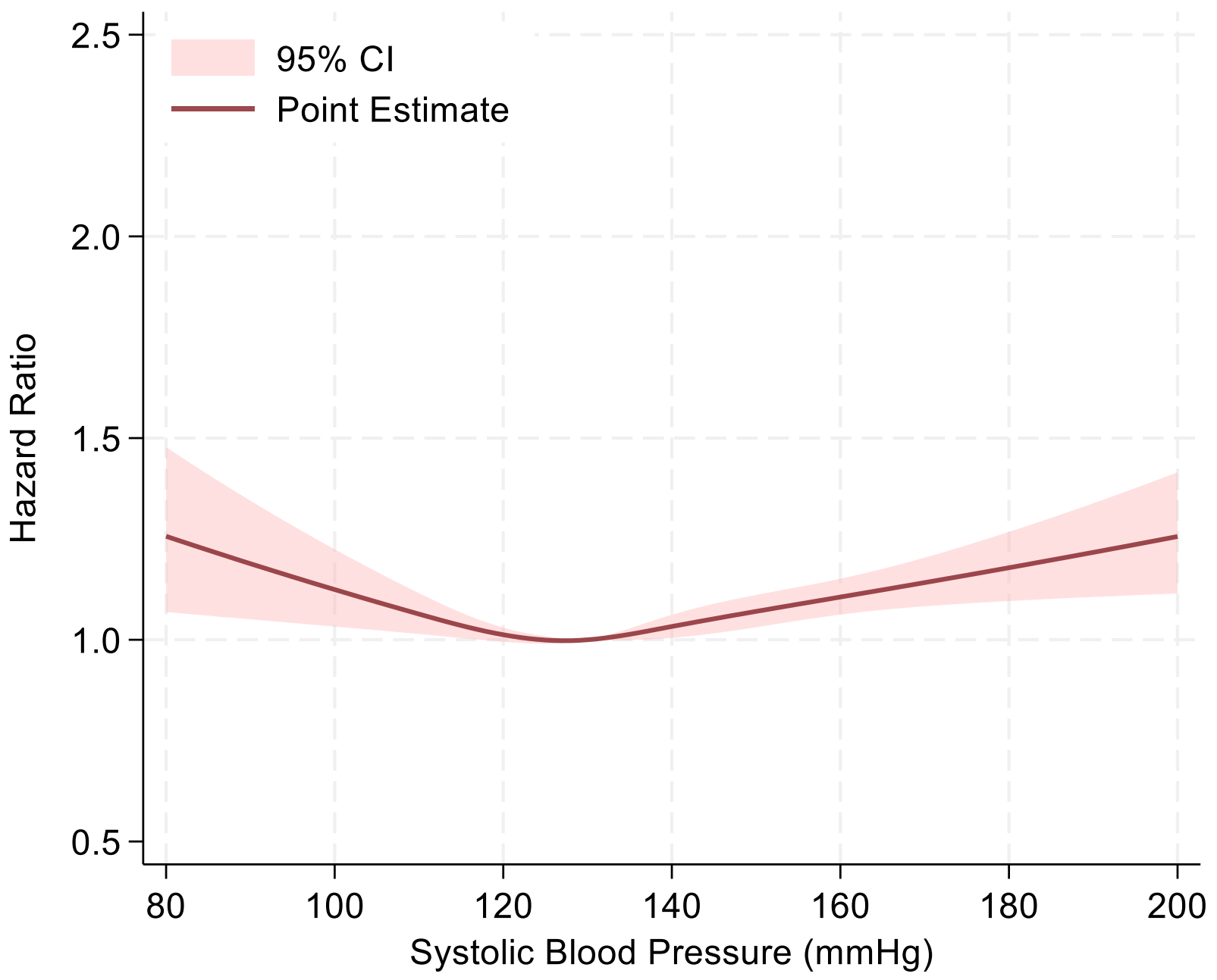

(B) Antihypertensive medication (-)

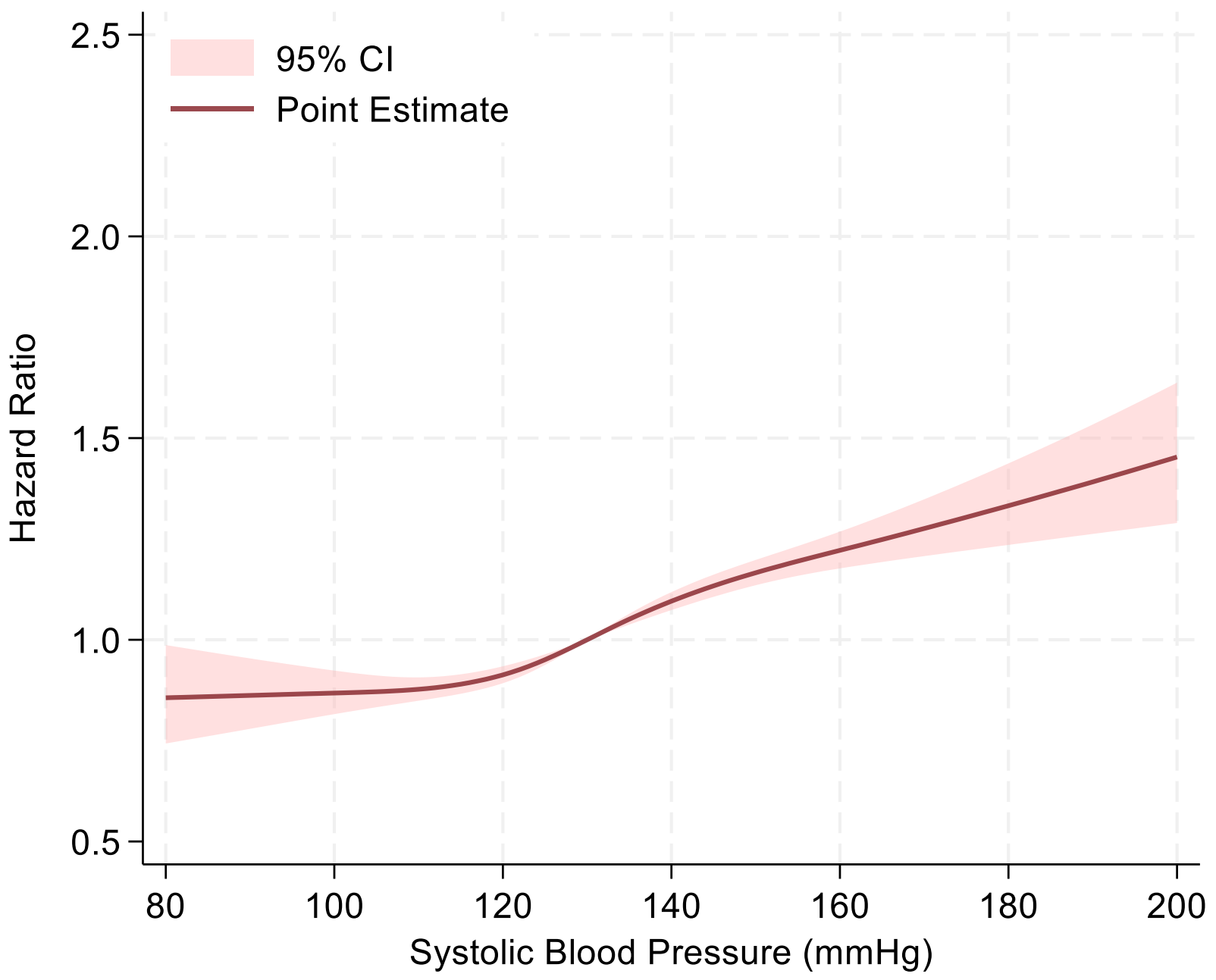

Supplementary Figure 8. Risk of developing heart failure according to systolic blood pressure using alternative outcome definition requiring at least two diagnosis codes within a 3-month period

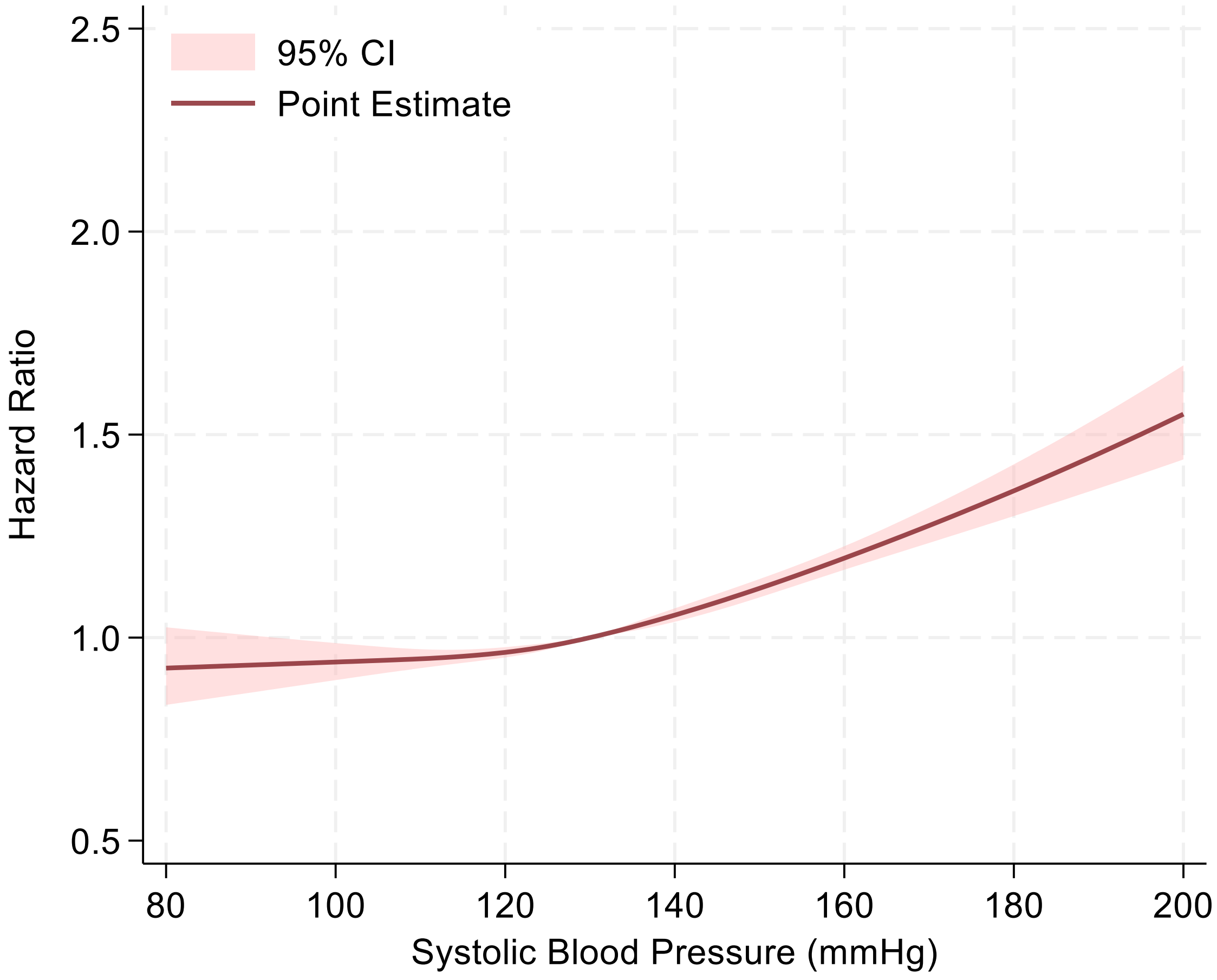

Supplementary Figure 9. Risk of developing composite cardiovascular disease according to systolic blood pressure using an alternative definition of diabetes

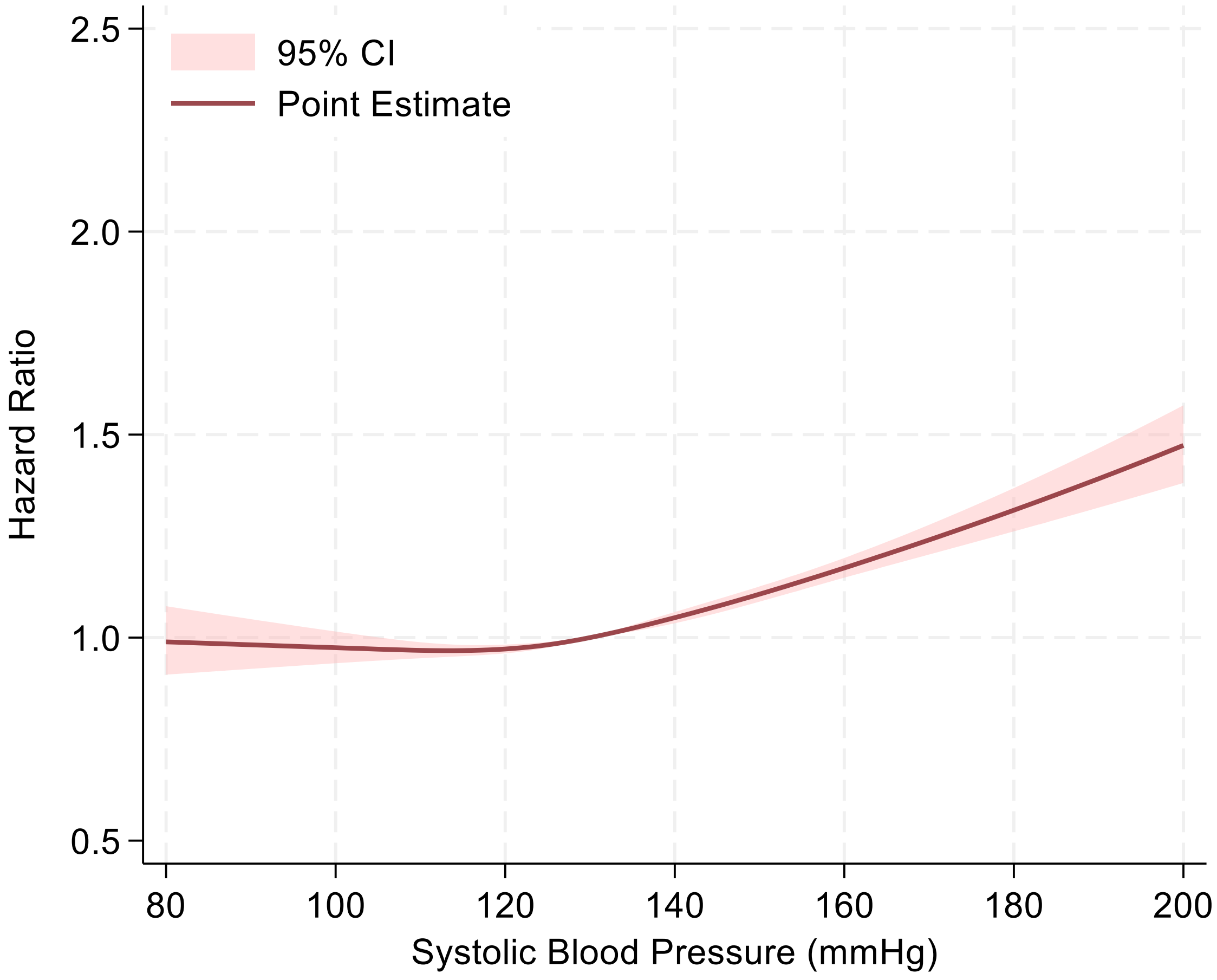

Supplementary Figure 10. Risk of developing composite cardiovascular disease according to systolic blood pressure using an alternative composite outcome definition excluding atrial fibrillation

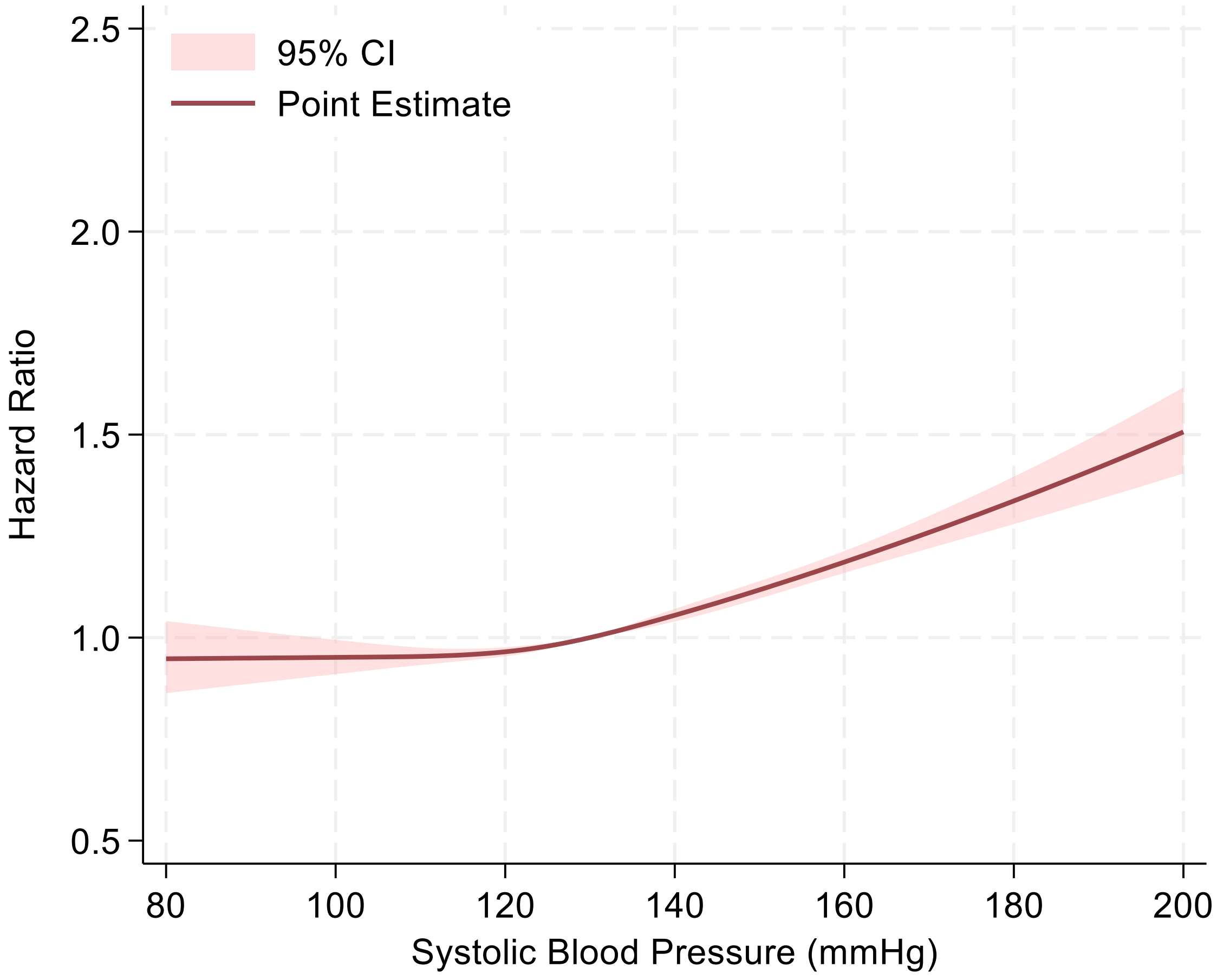

Supplementary Figure 11. Risk of developing composite cardiovascular disease according to systolic blood pressure using averaged blood pressure value

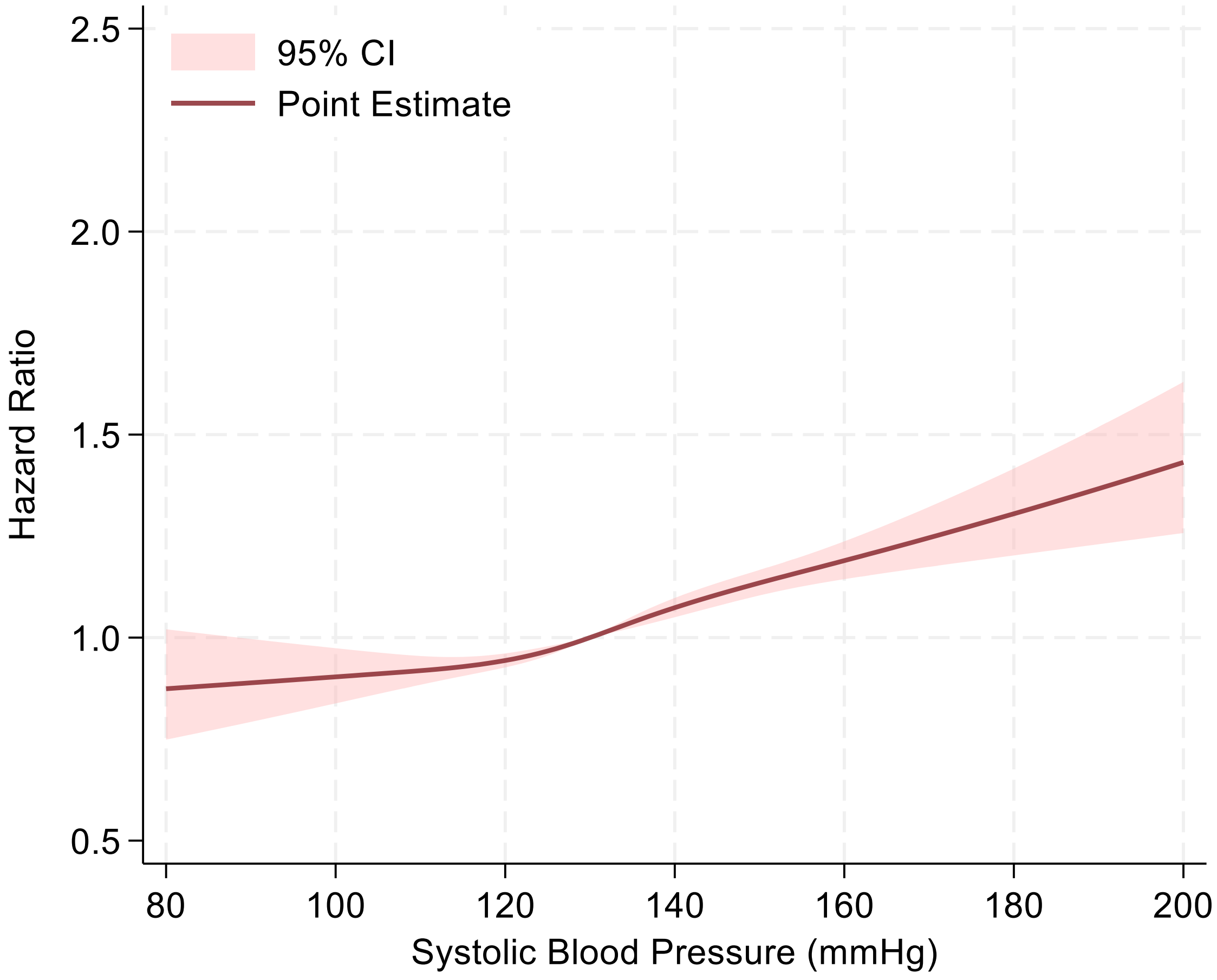

Supplementary Figure 12. Risk of developing composite cardiovascular disease according to systolic blood pressure among participants with eGFR < 60 mL/min/1.73 m^2 and negative or trace proteinuria at both the initial and one-year follow-up health checkups

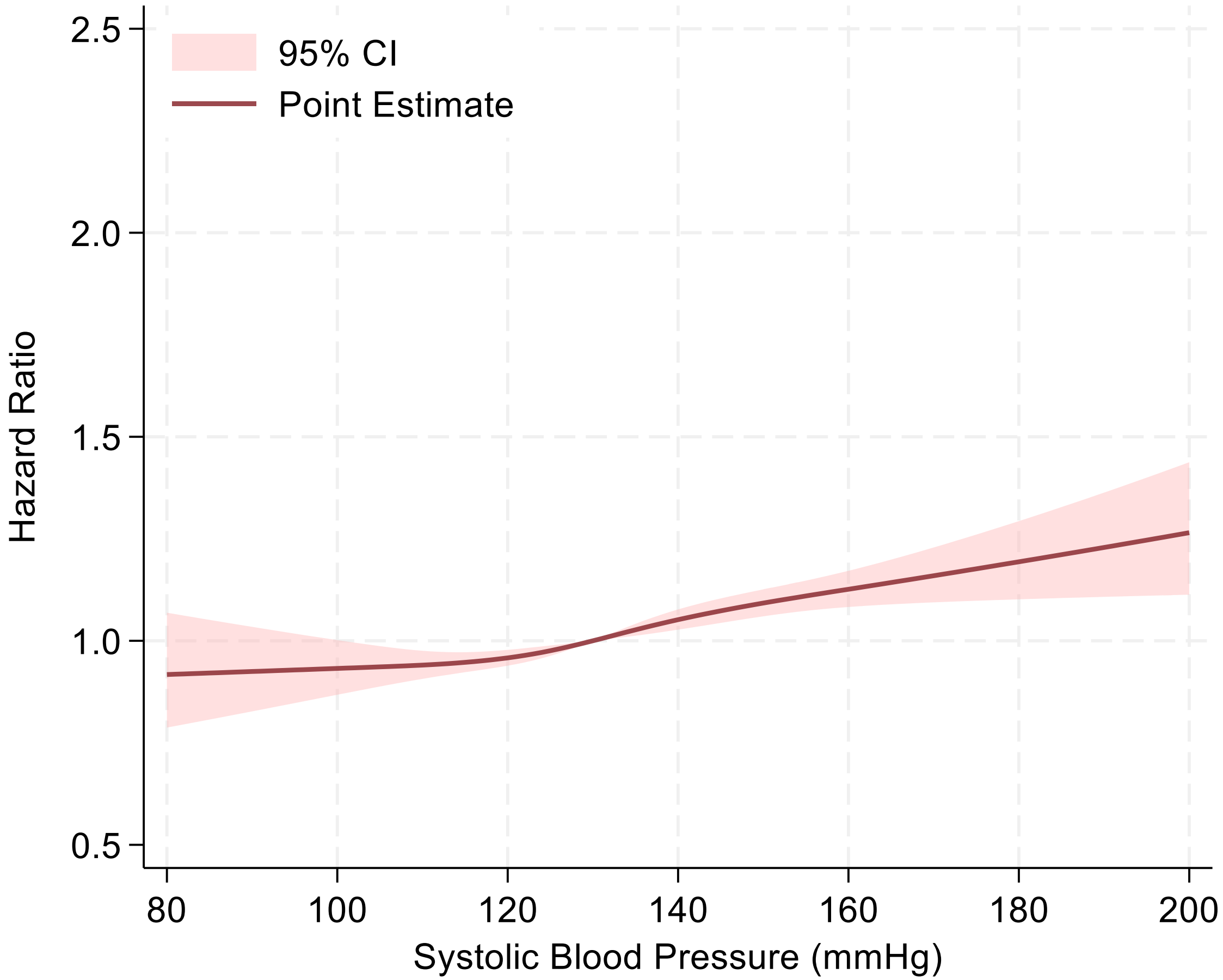

Supplementary Figure 13. Risk of developing composite cardiovascular disease according to systolic blood pressure among individuals with positive proteinuria

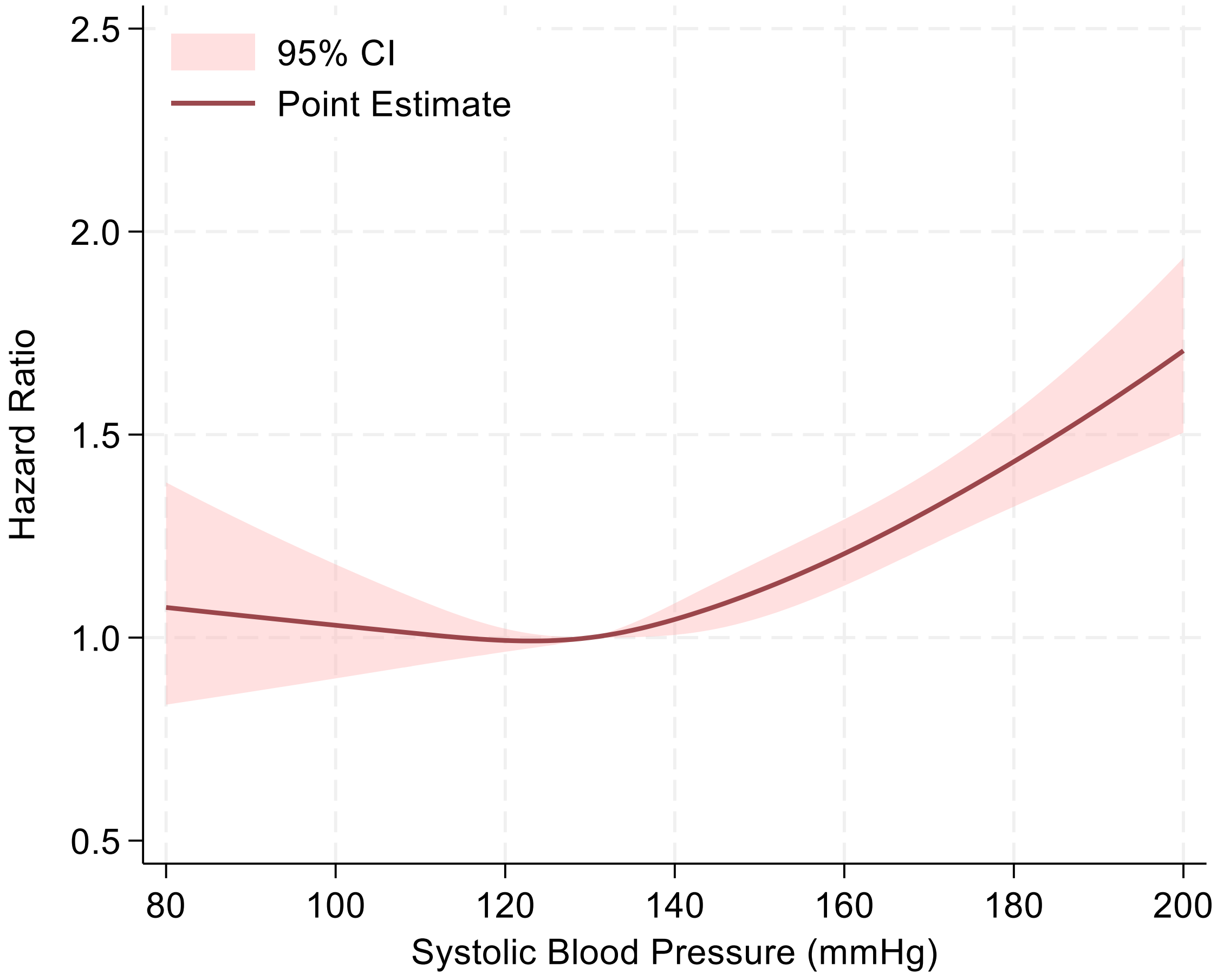

Supplement: Supplementary file 1 [file 41440_2026_2801_MOESM2_ESM.pdf]
