# Supplementary material for: Blood pressure and cardiovascular risk in adults with non-proteinuric chronic kidney disease without diabetes
Source: Hypertens Res. 2026 Sep 10;49(11):2999–3009. doi: 10.1038/s41440-026-02801-7 (PMC13736525; doi:10.1038/s41440-026-02801-7)
Supplement: Supplementary file 2 [file 41440_2026_2801_MOESM1_ESM.docx]

| **Supplementary Table 1. Baseline characteristics stratified by blood pressure category according to the 2017 ACC/AHA BP guidelines** | | | | |
| --- | --- | --- | --- | --- |
|  | Normal BP  (n=63,615) | Elevated BP  (n=50,961) | Stage 1 hypertension  (n=82,056) | Stage 2 hypertension  (n=90,606) |
| Age, years | 68 (63-73) | 71 (67-76) | 70 (66-76) | 71 (67-77) |
| Men, n (%) | 21,118 (33.2) | 18,169 (35.7) | 34,035 (41.5) | 38,701 (42.7) |
| BMI, kg/m^2^ | 21.9 (19.9-23.9) | 22.7 (20.8-24.8) | 23.2 (21.2-25.3) | 23.5 (21.4-25.6) |
| SBP, mmHg | 110 (104-115) | 124 (122-127) | 132 (130-136) | 148 (142-157) |
| DBP, mmHg | 66 (61-71) | 71 (67-75) | 80 (73-83) | 85 (78-92) |
| Antihypertensive medication, n (%) | 14,719 (23.1) | 19,120 (37.5) | 34,620 (42.2) | 40,442 (44.6) |
| Dyslipidemia, n (%) | 38,634 (60.7) | 33,830 (66.4) | 55,279 (67.4) | 60,924 (67.2) |
| Cigarette smoking, n (%) | 6,226 (9.8) | 4,170 (8.2) | 6,996 (8.5) | 7,554 (8.3) |
| Alcohol consumption, n (%) | 9,721 (15.3) | 9,236 (18.1) | 17,277 (21.1) | 20,695 (22.8) |
| HbA1c, % | 5.6 (5.4-5.8) | 5.6 (5.4-5.8) | 5.6 (5.4-5.8) | 5.6 (5.4-5.8) |
| LDL-C, mg/dL | 126 (106-147) | 125 (105-146) | 126 (106-147) | 128 (108-149) |
| HDL-C, mg/dL | 64 (53-77) | 62 (52-74) | 61 (51-73) | 61 (51-73) |
| Triglycerides, mg/dL | 93 (69-131) | 101 (75-141) | 105 (77-147) | 109 (79-152) |
| eGFR, ml/min per 1.73 m^2^ | 55.3 (51.3-57.9) | 54.9 (50.6-57.7) | 54.9 (50.7-57.7) | 54.7 (50.5-57.7) |
| ***Proteinuria, n (%)*** |  |  |  |  |
| Negative | 57,462 (90.3) | 45,789 (89.9) | 72,989 (89.0) | 79,309 (87.5) |
| Trace | 6,153 (9.7) | 5,172 (10.1) | 9,067 (11.0) | 11,297 (12.5) |
| Data are reported as medians (interquartile range) or numbers (percentage), where appropriate.  ACC, American college of cardiology; AHA, American heart association; BMI, body mass index; BP, blood pressure; SBP, systolic blood pressure; DBP, diastolic blood pressure; LDL-C, low-density lipoprotein cholesterol; HDL-C, high-density lipoprotein cholesterol; eGFR, estimated glomerular filtration rate. | | | | |

| **Supplementary Table 2. Blood pressure and cardiovascular events (continuous variable)** | | | | |
| --- | --- | --- | --- | --- |
|  | SBP per 10 mmHg increase | | DBP per 10 mmHg increase | |
|  | Hazard Ratio | P value | Hazard Ratio | P value |
| Composite events |  |  |  |  |
| Model 1 | 1.12 (1.11-1.12) | <0.001 | 0.99 (0.98-1.00) | 0.137 |
| Model 2 | 1.05 (1.05-1.06) | <0.001 | 1.06 (1.06-1.07) | <0.001 |
| Model 3 | 1.05 (1.04-1.05) | <0.001 | 1.06 (1.05-1.07) | <0.001 |
| Model 1 is unadjusted. Model 2 includes adjustment for age and sex. Model 3 includes adjustment for age, sex, body mass index, antihypertensive medication, hemoglobin A1c, dyslipidemia, cigarette smoking, alcohol consumption, estimated glomerular filtration rate, and proteinuria status. | | | | |

| **Supplementary Table 3. Subgroup analysis of the association between blood pressure category according to the 2017 ACC/AHA BP guidelines and cardiovascular events** | | | | |
| --- | --- | --- | --- | --- |
|  | Normal BP | Elevated BP | Stage 1 hypertension | Stage 2 hypertension |
| Men | | | | |
| Number | 21,118 | 18,169 | 34,035 | 38,701 |
| Events | 2,726 | 2,758 | 4,882 | 6,552 |
| Incidence | 411.7 (396.5-427.5) | 510.1 (491.4-529.5) | 483.1 (469.7-496.8) | 595.3 (581.0-609.9) |
| Model 1 | 1 [Reference] | 1.24 (1.17-1.30) | 1.17 (1.12-1.23) | 1.44 (1.38-1.51) |
| Model 2 | 1 [Reference] | 1.04 (0.99-1.10) | 1.06 (1.01-1.11) | 1.24 (1.18-1.29) |
| Model 3 | 1 [Reference] | 1.02 (0.97-1.08) | 1.03 (0.98-1.08) | 1.20 (1.14-1.25) |
| Women | | | | |
| Number | 42,497 | 32,792 | 48,021 | 51,905 |
| Events | 4,130 | 4,273 | 6,749 | 8,478 |
| Incidence | 314.2 (304.8-323.9) | 436.8 (423.9-450.1) | 477.6 (466.3-489.1) | 573.3 (561.2-585.6) |
| Model 1 | 1 [Reference] | 1.39 (1.33-1.45) | 1.52 (1.46-1.58) | 1.83 (1.76-1.90) |
| Model 2 | 1 [Reference] | 1.07 (1.03-1.12) | 1.16 (1.12-1.21) | 1.26 (1.21-1.31) |
| Model 3 | 1 [Reference] | 1.04 (1.00-1.09) | 1.11 (1.06-1.15) | 1.19 (1.15-1.24) |
| Age ≥ 70 years | | | | |
| Number | 27,491 | 29,956 | 45,941 | 55,407 |
| Events | 4,375 | 5,310 | 8,412 | 11,213 |
| Incidence | 606.7 (589.0-624.9) | 666.5 (648.8-684.6) | 695.5 (680.8-710.6) | 784.4 (770.0-799.0) |
| Model 1 | 1 [Reference] | 1.09 (1.05-1.14) | 1.14 (1.10-1.18) | 1.29 (1.25-1.34) |
| Model 2 | 1 [Reference] | 1.04 (1.00-1.08) | 1.08 (1.04-1.12) | 1.18 (1.14-1.22) |
| Model 3 | 1 [Reference] | 1.01 (0.97-1.05) | 1.05 (1.01-1.09) | 1.14 (1.10-1.18) |
| Age < 70 years | | | | |
| Number | 36,124 | 21,005 | 36,115 | 35,199 |
| Events | 2,481 | 1,721 | 3,219 | 3,817 |
| Incidence | 197.6 (190.0-205.5) | 238.3 (227.3-249.8) | 265.1 (256.1-274.4) | 331.9 (321.6-342.6) |
| Model 1 | 1 [Reference] | 1.21 (1.13-1.28) | 1.34 (1.28-1.42) | 1.69 (1.60-1.77) |
| Model 2 | 1 [Reference] | 1.10 (1.03-1.17) | 1.20 (1.14-1.26) | 1.46 (1.39-1.54) |
| Model 3 | 1 [Reference] | 1.05 (0.99-1.12) | 1.12 (1.06-1.18) | 1.35 (1.28-1.42) |
| Proteinuria trace | | | | |
| Number | 6,153 | 5,172 | 9,067 | 11,297 |
| Events | 865 | 962 | 1,604 | 2,390 |
| Incidence | 459.7 (430.1-491.4) | 622.0 (583.9-662.6) | 599.1 (570.5-629.1) | 743.4 (714.2-773.8) |
| Model 1 | 1 [Reference] | 1.35 (1.23-1.48) | 1.30 (1.20-1.41) | 1.62 (1.50-1.75) |
| Model 2 | 1 [Reference] | 1.08 (0.99-1.18) | 1.09 (1.00-1.18) | 1.26 (1.16-1.36) |
| Model 3 | 1 [Reference] | 1.05 (0.96-1.15) | 1.05 (0.97-1.15) | 1.21 (1.12-1.31) |
| Proteinuria negative | | | | |
| Number | 57,462 | 45,789 | 72,989 | 79,309 |
| Events | 5,991 | 6,069 | 10,027 | 12,640 |
| Incidence | 335.0 (326.6-343.6) | 444.8 (433.8-456.2) | 465.1 (456.1-474.3) | 559.8 (550.1-569.6) |
| Model 1 | 1 [Reference] | 1.33 (1.28-1.37) | 1.39 (1.34-1.43) | 1.67 (1.62-1.72) |
| Model 2 | 1 [Reference] | 1.06 (1.03-1.10) | 1.13 (1.09-1.17) | 1.26 (1.22-1.30) |
| Model 3 | 1 [Reference] | 1.03 (0.99-1.07) | 1.08 (1.05-1.12) | 1.20 (1.16-1.24) |
| eGFR ≥ 54.9 mL/min/1.73m^2^ | | | | |
| Number | 33,561 | 25,260 | 40,962 | 43,852 |
| Events | 2,940 | 2,893 | 4,835 | 6,260 |
| Incidence | 274.7 (264.9-284.8) | 379.0 (365.4-393.1) | 393.0 (382.1-404.3) | 495.0 (482.9-507.5) |
| Model 1 | 1 [Reference] | 1.38 (1.31-1.45) | 1.43 (1.37-1.50) | 1.80 (1.73-1.88) |
| Model 2 | 1 [Reference] | 1.09 (1.03-1.14) | 1.14 (1.09-1.20) | 1.32 (1.27-1.38) |
| Model 3 | 1 [Reference] | 1.05 (1.00-1.11) | 1.09 (1.04-1.14) | 1.25 (1.19-1.30) |
| eGFR < 54.9 mL/min/1.73m^2^ | | | | |
| Number | 30,054 | 25,701 | 41,094 | 46,754 |
| Events | 3,916 | 4,138 | 6,796 | 8,770 |
| Incidence | 432.2 (418.8-445.9) | 547.6 (531.1-564.5) | 569.4 (556.0-583.1) | 666.9 (653.1-681.0) |
| Model 1 | 1 [Reference] | 1.27 (1.21-1.32) | 1.32 (1.27-1.37) | 1.54 (1.49-1.60) |
| Model 2 | 1 [Reference] | 1.05 (1.01-1.10) | 1.11 (1.07-1.16) | 1.22 (1.17-1.27) |
| Model 3 | 1 [Reference] | 1.02 (0.98-1.07) | 1.07 (1.03-1.12) | 1.17 (1.13-1.22) |
| Antihypertensive medication (+) | | | | |
| Number | 14,719 | 19,120 | 34,620 | 40,442 |
| Events | 2,759 | 3,580 | 6,251 | 8,073 |
| Incidence | 647.0 (623.3-671.5) | 644.4 (623.6-665.8) | 625.0 (609.7-640.7) | 704.7 (689.5-720.2) |
| Model 1 | 1 [Reference] | 0.99 (0.95-1.05) | 0.97 (0.92-1.01) | 1.09 (1.04-1.14) |
| Model 2 | 1 [Reference] | 0.91 (0.86-0.95) | 0.93 (0.89-0.98) | 0.99 (0.95-1.04) |
| Model 3 | 1 [Reference] | 0.91 (0.87-0.96) | 0.94 (0.90-0.99) | 1.01 (0.96-1.05) |
| Antihypertensive medication (-) | | | | |
| Number | 48,896 | 31,841 | 47,436 | 50,164 |
| Events | 4,097 | 3,451 | 5,380 | 6,957 |
| Incidence | 264.3 (256.3-272.5) | 358.2 (346.4-370.4) | 377.9 (368.0-388.2) | 485.2 (473.9-496.7) |
| Model 1 | 1 [Reference] | 1.35 (1.29-1.42) | 1.43 (1.37-1.49) | 1.84 (1.77-1.91) |
| Model 2 | 1 [Reference] | 1.10 (1.05-1.15) | 1.17 (1.12-1.21) | 1.38 (1.33-1.43) |
| Model 3 | 1 [Reference] | 1.10 (1.05-1.15) | 1.16 (1.11-1.21) | 1.36 (1.31-1.42) |
| Model 1 is unadjusted. Model 2 includes adjustment for age and sex. Model 3 includes adjustment for age, sex, body mass index, antihypertensive medication, hemoglobin A1c, dyslipidemia, cigarette smoking, alcohol consumption, estimated glomerular filtration rate, and proteinuria status. For the subgroup analyses stratified by sex, proteinuria, and antihypertensive medication, we excluded the stratifying variable (sex, proteinuria, or antihypertensive medication) from the covariates.  ACC, American college of cardiology; AHA, American heart association; BMI, body mass index; BP, blood pressure | | | | |

| **Supplementary Table 4. Baseline characteristics stratified by missing status of covariates** | | | |
| --- | --- | --- | --- |
|  | Non-missing  (n=287,238) | Missing  (n=96,479) | Missing data, n |
| Age, years | 70 (66-76) | 77 (76-81) | 0 |
| Men, n (%) | 112,023 (39.0) | 36,721 (38.1) | 0 |
| BMI, kg/m^2^ | 22.9 (20.9-25.0) | 22.8 (20.8-24.9) | 0 |
| SBP, mmHg | 130 (120-142) | 133 (123-144) | 0 |
| DBP, mmHg | 76 (69-83) | 74 (68-81) | 0 |
| Antihypertensive medication, n (%) | 108,901 (37.9) | 48,039 (49.8) | 0 |
| Dyslipidemia, n (%) | 188,667 (65.7) | 62,813 (65.1) | 0 |
| Cigarette smoking, n (%) | 24,946 (8.7) | 1,374 (6.6) | 75,505 |
| Alcohol consumption, n (%) | 56,929 (19.8) | 157 (18.0) | 95,607 |
| HbA1c, % | 5.6 (5.4-5.8) | 5.6 (5.4-5.8) | 0 |
| LDL-C, mg/dL | 126 (106-147) | 121 (102-141) | 0 |
| HDL-C, mg/dL | 62 (51-74) | 62 (51-73) | 0 |
| Triglycerides, mg/dL | 103 (75-144) | 102 (76-141) | 0 |
| eGFR, ml/min per 1.73 m^2^ | 54.9 (50.8-57.7) | 53.5 (48.5-57.0) | 0 |
| ***Proteinuria, n (%)*** |  |  | 0 |
| Negative | 255,549 (89.0) | 83,009 (86.0) | - |
| Trace | 31,689 (11.0) | 13,470 (14.0) | - |
| Data are reported as medians (interquartile range) or numbers (percentage), where appropriate. Missing indicates missing data on cigarette smoking and/or alcohol consumption.  BMI, body mass index; SBP, systolic blood pressure; DBP, diastolic blood pressure; LDL-C, low-density lipoprotein cholesterol; HDL-C, high-density lipoprotein cholesterol; eGFR, estimated glomerular filtration rate. | | | |

| **Supplementary Table 5. Blood pressure category according to the 2017 ACC/AHA BP guidelines and cardiovascular events** **in multiple imputations** | | | | |
| --- | --- | --- | --- | --- |
|  | Normal BP  (n=78,895) | Elevated BP  (n=68,905) | Stage 1 hypertension  (n=110,200) | Stage 2 hypertension  (n=125,717) |
| Composite |  |  |  |  |
| Events | 8,878 | 9,503 | 15,364 | 20,211 |
| Incidence | 388.8 (380.8-396.9) | 508.2 (498.1-518.5) | 516.1 (508.0-524.3) | 622.8 (614.3-631.5) |
| Model 1 | 1 [Reference] | 1.30 (1.27-1.34) | 1.33 (1.29-1.36) | 1.60 (1.56-1.64) |
| Model 2 | 1 [Reference] | 1.05 (1.02-1.08) | 1.09 (1.06-1.12) | 1.22 (1.19-1.25) |
| Model 3 | 1 [Reference] | 1.02 (0.99-1.05) | 1.04 (1.02-1.07) | 1.16 (1.13-1.19) |
| Multiple imputation by chained equations was used to handle missing data on cigarette smoking and alcohol consumption. Twenty imputed datasets were generated. Model 1 is unadjusted. Model 2 includes adjustment for age and sex. Model 3 includes adjustment for age, sex, body mass index, antihypertensive medication, hemoglobin A1c, dyslipidemia, cigarette smoking, alcohol consumption, estimated glomerular filtration rate, and proteinuria status.  ACC, American college of cardiology; AHA, American heart association; BMI, body mass index; BP, blood pressure | | | | |
